# Population-level analysis of chronic disease multimorbidity at older ages across time using mixed graphical models

**DOI:** 10.1186/s13690-026-01886-3

**Published:** 2026-03-17

**Authors:** Linh Hoang Khanh Dang, Nicola Caranci, Daniela Fortuna, Giulia Roli, Rosella Rettaroli, Rossella Miglio

**Affiliations:** 1 Department of Statistical Sciences, University of Bologna, Bologna, Italy; 2 Department of Innovation in Health and Social Care, Emilia-Romagna Region, Bologna, Italy

**Keywords:** Multimorbidity, Chronic diseases, Older ages, Mixed graphical model, Network analysis, Longitudinal, Public health

## Abstract

**Background:**

As populations are aging globally and healthcare systems are transitioning from being centred around single disease to patient-centred approach, precise and flexible identification of the multimorbidity patterns allows sound measures to prevent adverse health outcomes and better allocate healthcare resources.

**Methods:**

Combining probabilistic approach of graphical model and intuitive visibility of network analysis, we use administrative health data of individuals aged 50 and above residing in Emilia-Romagna region (northern Italy) in 2011 and followed up to 2019 (*N* = 1,010,571) to investigate multimorbidity patterns and their impact across time.

**Results:**

Four consistent multimorbidity patterns are identified across sex, across age groups (50–59, 60–69, 70–79, 80 +), and across time-points (2011, 2016 and 2019), which consists of cardiovascular, neuropsychiatric, respiratory-digestive and metabolic-pain pattern. This finding suggests plausible existence of stable population multimorbidity structure for public health monitoring.

We also bring evidence of the better performance of multimorbidity patterns in mortality modelling compared to traditional methods. While neuropsychiatric pattern is the leading group causing mortality at older ages, higher education and living in suburban areas provide consistent protective effect against mortality, unplanned hospitalization and length of stay for all sexes and age groups.

We focus on the gatekeeper diseases as potential targets for intervention. They are not necessarily the diseases with highest prevalence, but we demonstrate that an early detection of these diseases can contribute to improving three different health outcomes at older ages.

**Conclusion:**

For each sex and age group above 50, we provide systematically the network of chronic diseases, the corresponding multimorbidity patterns, and the estimated impacts of multimorbidity, and thus a comprehensive understanding of multimorbidity at older ages. The observed consistent multimorbidity structure at population level and the identification of target diseases for intervention that goes beyond the prevalence-based approach can open new doors for more directions shaping public health policy.

**Supplementary Information:**

The online version contains supplementary material available at 10.1186/s13690-026-01886-3.

**Table:** 

Texbox 1. Contributions to the literature
• The paper documents the consistency of the network-based chronic disease multimorbidity patterns in older adults across time points.
• This population-level temporal stability in multimorbidity structure provides a reliable basis for examining longitudinal impacts.
• Incorporating network-based multimorbidity patterns improves the performance of mortality models.
• Neuropsychiatric pattern is the leading group behind mortality at older ages; strongest effects are found as early as ages 50–59 for both sexes.
• Network metrics help identify public health intervention targets that otherwise might be overlooked under prevalence-based approaches.

## Background

The increase of global human life expectancy, soaring from around 25–35 years to 73.3 years in our present days, has been one of the most remarkable successes of humankind in using science and technology to improve public health [1]. On average, people are living longer, but also possibly in poor health and increasing dependence, which could lead to further consequences to the society when the conditions in education, wealth, infrastructure and technology have not yet been secured to accommodate the needs of older people [2]. Among these complications, the growing proportion of individuals that are affected with two or more diseases, often considered as patients with multimorbidity, constitutes particular concerns [3]. Even though the risk of dying from chronic diseases dropped in many countries, less is known about the impacts of multimorbidity of chronic diseases [4]. However, traditional methods to take into account multimorbidity (e.g., total number of diseases, co-morbidity indexes) are oftentimes aggregated metrics and have limitations in information that they can deliver. Meanwhile, if it is reasonable to think that the chronic conditions cluster non-randomly under common risk factors into coherent groups that we can identify, mapping these clusters will allow us not only to summarize the multimorbidity conditions within a population, but also constitutes great opportunities to ”uncover the mechanisms for diseases accumulation, develop treatments and reconfigure the healthcare system to better cater for the patients’ needs” [5].

In the literature, three commonly-used approaches to identify clusters of chronic diseases include explanatory factor analysis [6], clustering (both cluster analyses of diseases [7] and cluster analysis of patients [8], and network analysis [9]. Each approach represents however limitations. Factor analysis helps identify the co-occurrence between diseases, but it allows one disease to belong simultaneously to several groupings and some diseases might appear virtually in all patterns, which complicates the interpretation of the identified clusters. In cluster analyses where soft clustering techniques such as fuzzy c-means are used, we encounter the same limitations. Moreover, in clustering analyses, it is not possible to incorporate the strength of relationship between the individual diseases within each cluster. As for network analysis, although it offers powerful advantage of visualizing the strength of associations between diseases and has advanced to become even a data format rendered for research purpose [10], such associations often convey only descriptive value rather than predictive power. In addition, depending on the choice of association measures (e.g., Pearson’s phi, Jaccard index, relative risk, lift), the results can be different, i.e., different network maps can be produced using the same set of data, and thus multimorbidity network analyses are sensitive to the disease co-occurrence measurement [11].

Given these limitations, the combination of mixed graphical model and network analysis constitutes a reliable method to mitigate the challenges previously mentioned [12]. Compared to the original study, we apply this approach on the data of residents aged 50 and above at census 2011 in Emilia-Romagna region (northern Italy), with several strengthened points.

Firstly, a more extensive period of observation from 2011 to 2019 allows for better capture of the chronic conditions that might take time to manifest, which can bring more insights than relying on one-year observations. Secondly, a longitudinal dataset of a population in the region gives opportunities to evaluate the consistency of the empirically identified patterns at three different time-points (in 2011, 2016 and 2019), which has not been done in the literature to our best knowledge. In fact, although there are evidences that several similar clusters of chronic diseases can be found across existing studies, namely mental health and cardio-metabolic conditions [13], less is known about the consistence of such empirically-identified multimorbidity patterns across time, gender and age groups. Finally, since further research is still needed to illustrate the use of multimorbidity patterns in practice, we assess the mortality risks and other health outcomes using the identified patterns and other metrics from the chronic diseases network in monitoring the multimorbidity situation of a population at a given time, shedding light on the role of multimorbidity in health conditions at older ages by sex and age group.

## Data and methods

### Data

Emilia-Romagna region is located in northern Italy, consists of nine provinces spanning over 22,000 km^2^ of urban, suburban and rural communities. This region is the residence place for more than 4.4 millions habitants between 2011 and 2019. The data sources for the study include the census data in 2011, the population register, and the healthcare administrative data from 2011 to 2019 in the region, which are linked together using a record linkage based on a pseudonymisation process and an attribution of a double personal identification key.

By combining the census data with population register, we are able to identify exhaustively the individuals aged 50 and above (*N* = 1,377,325). From census data, we also obtain other covariates including sex, date of birth (and date of death, if the individual passed away during observation period), civil status, education level and place of residence for each individual. Our study population is a closed-cohort, the individuals who were present at census date but moved out of the region during follow-up period are right-censored, the individuals who moved into the region after census date are excluded. Only the individuals who were diagnosed with at least two chronic diseases during observation period are included in the study. Our final population include 1,010,571 individuals (557,562 are females and 453,009 are males). We stratify our population by sex and by age group following age at census (50–59, 60–69, 70–79, 80 +), resulting in eight subgroups. We conduct analyses systematically for each subgroup, controlling therefore systematically for sex and age.

The Table 1 presents the characteristics of the study population. The median number of diseases increases with ages, suggesting an accumulation of chronic conditions as the individual survives to older ages. The level of education saw improvements in younger cohorts for both females and males. For cohorts aged 70 and above, a majority of multimorbid patients obtained no education or primary education. Smaller cities or suburban areas are the main location of residence across all age groups for both sexes. The death counts, the median number of unplanned hospitalization and the median length of stay in hospitals increase with age cohorts.

**Table 1:** Descriptive statistics of female (F) and male (M) populations aged 50 and above with multimorbidity stratified by age-group cohort recorded at 2011 census in Romagna-Emilia region

	F50-59, F60-69, F70-79	F80+	M50-59, M60-69	M70-79	M80 +
Observations number	111,521,146,692,158,861	140,488	99,962,137,318	135,231	80,498
Number of diseases	3 (IQR 2–4) 4 (IQR 3–5) 5 (IQR 3–7)	6 (IQR 4–8)	3 (IQR 2–4) 4 (IQR 3–6)	5 (IQR 3–7)	6 (IQR 4–8)
2	36.2823.4913.92	9.55	35.5321.63	11.83	7.07
3	26.0622.6816.52	12.15	24.9220.51	14.09	9.94
4 +	37.6653.8369.56	78.30	39.5557.86	74.08	82.99
Total (%)	100.00100.00100.00	100.00	100.00100.00	100.00	100.00
Education					
No education/Primary	15.1948.2172.72	82.39	12.7135.92	60.75	71.63
Lower second	48.1332.3617.24	10.45	47.6836.02	23.15	16.10
Higher second	24.7312.437.16	5.04	28.0018.56	10.50	7.35
University	11.957.002.87	2.13	11.619.50	5.60	4.92
Total (%)	100.00100.00100.00	100.00	100.00100.00	100.00	100.00
Urban/rural residence					
Cities	34.0736.9238.73	39.01	32.8834.45	36.22	37.38
Smaller cities/suburban	44.5943.2841.48	39.14	44.6643.75	42.43	40.31
Rural	21.3319.8019.78	21.84	22.4521.80	21.35	22.31
Total (%)	100.00100.00100.00	100.00	100.00100.00	100.00	100.00
Health outcomes					
Death counts	5,60,914,18,241,431,100,897		8,36,521,93,450,48,163,325		
Number of unplanned hospitalizations	1 (IQR 0–2) 1 (IQR 0–2) 1 (IQR 0–3) 2 (IQR 1–3)		1 (IQR 0–2) 1 (IQR 0–2)2 (IQR 1–3)2 (IQR 1–4)		
Length of stay (days)	1 (IQR 0–5) 1 (IQR 0–9) 6 (IQR 0–22) 15 (IQR 4–33)		1 (IQR 0–9) 3 (IQR 0–15) 10 (IQR 1—29) 18 (IQR 6–36)		

The number of diseases is the total number of chronic conditions that individuals are diagnosed with across the observation period from 2011 to 2019. The level of education and the rural/urban residence are retrieved at baseline 2011 census. IQR is the interquartile range

The chronic diseases for each individual are classified in ICD-9-CM and grouped into 33 main chronic conditions using the coding algorithm by [14] for Emilia-Romagna region based on ten different health databases (e.g., hospital discharge, community hospital discharge, local pharmaceutical assistance, home care, residential assistance for elderly people). For the stability of statistical analyses, we excluded diseases with pooled prevalence less than 1%. Our final set consists of 30 chronic diseases, neoplasms consists of both uncertain behavior and malignant cancers. Other individual medical conditions such as unplanned hospitalizations and length of stay are retrieved from hospital administrative data.

The socioeconomic characteristics incorporated in our analyses include the education level and the urbanity of individual’s residence place. The education level is divided into four categories of highest attained degree, including no education/primary education, lower second education, higher second education and university. The urbanity is assessed for each municipality and divided into cities, small cities/suburban areas and rural areas, according to EU Regulation 2017/2391. These variables are time-invariant and are collected at the beginning of the observation period.

### Methods

The objectives of this study are three-fold: (1) estimate the chronic diseases network structure and identify the corresponding multimorbidity patterns, (2) evaluate the consistency of these patterns across time-points and subpopulations, estimate their impacts on mortality, and (3) identify possible target diseases for public health intervention and assess their impacts on different health outcomes. All analyses are conducted using R software version 4.4.2. More of the results can also be found in the supplementary files.

#### Estimation of the chronic diseases network structure and identification of the multimorbidity patterns

For the first objective, we use mixed graphical model to estimate the network structure of the chronic conditions for each sub-population [12]. This approach allows us to obtain, as output, a network of chronic diseases where the edge linking the nodes of chronic diseases represents the pairwise partial correlation between them. In other words, the resulted network represents a system of unique interaction between two chronic diseases after controlling for all other chronic diseases, known as a network of conditional independence associations between diseases [15, 16].

We apply communities detection algorithm to these estimated networks and cluster the chronic diseases nodes into separate groupings, where each disease can belong only to one pattern (i.e., hard clustering) [17]. Rather than choosing arbitrarily a clustering algorithm among many possibilities, we apply systematically 7 most-widely used communities detection algorithms in the literature (edge-between, fast greedy, Louvain, walkstrap, spinglass, optimal, label propagation) and select the algorithm most suitable to our dataset based on modularity score [18–20]. The higher the modularity score, the denser connections there are between nodes within a cluster and the sparser connections between nodes of different clusters, and thus the better the algorithm performs. We apply the resulted best-performed algorithm for 2 sexes, 4 age-groups and at 3 time-points (in 2011, 2016 and 2019), resulting in 24 estimated networks.

It is also noteworthy that by following the method detailed above, even though each disease belongs only to one pattern, each individual can belong to multiple patterns simultaneously, and there might be a need to assign one unique multimorbidity pattern to each patient for modeling purpose. This task can be thought of assigning the pattern that is most substantial for each patient, but the rationale of how a pattern can be considered as ”substantial” varies depending on the purpose of the study. In this paper, we focus on putting forward the diseases (and thus their corresponding pattern) whose management or prevention can contribute to reduce the burden of outcomes for individuals and society. From network perspective, we define these diseases as the ones that hold strong associations with other diseases and are likely to be caused by other diseases. We rely on two metrics, the strength centrality and the in-degree measurements respectively, to quantify these characteristics.

We compute the strength centrality from the network of chronic diseases estimated by mixed graphical model and the in-degree from the Bayesian network of chronic diseases estimated using the hill-climbing algorithm. We obtain the score for each disease by summing their strength centrality and their in-degree within each network. If the individual belongs to only one pattern, we assign that pattern for the individual. If the individual belongs to more than one pattern, we assign the pattern that has a higher number of diseases. If the number of diseases between different patterns of an individual is equal, we assign the individual to the pattern that has the highest score that is computed as detailed above.

#### Estimation of the multimorbidity impacts on mortality using patterns of chronic diseases

For the second objective, we apply a set of two Cox models, using the total number of chronic diseases in the first model and the developed multimorbidity patterns as time-dependent predictor in the second model. We use the Akaike Information Criterion (AIC) to compare the performance between two models in each sub-population and evaluate the performance of using the multimorbidity patterns in assessing the mortality of multimorbid patients. Afterwards, we apply the full models including the multimorbidity patterns and the total number of diseases on the dataset of 8 subgroups (hence controlled for age-group and gender), controlled for the level of education and the urban/rural location of residence.

#### Identification of the target diseases from chronic diseases network structure

The chronic diseases network does not only serve as the base to establish multimorbidity patterns, its structure can also lend insights for practical application. In this study, we identify the potential target diseases for public health intervention using betweenness centrality. We term the diseases with the highest betweenness centrality the ”gatekeeper diseases” for their role in linking otherwise unconnected diseases or groups of diseases within the network, and assess the impact of having these diseases in 2011 on health outcomes observed until 2019. These health outcomes include the mortality, the number of hospitalization and the length of hospitalization. All analyses are done within the generalized linear models with the appropriate assumed distribution for each outcome variable (i.e., logistic regression for mortality, Poisson regression for the number of hospitalization accounting for over-dispersion, linear regression on the logarithm of length of stay to account for skewness of the data), systematically for all sub-populations stratified by sex and age-group cohort (aged 50–59, 60–69, 70–79, 80 + at 2011 census).

## Results

### Identification of the multimorbidity patterns at older ages

The chronic diseases network is estimated using mixed graphical model for each of 24 sub-populations, presenting the estimated pairwise partial correlations between chronic diseases. Based on AIC, we found that the optimal algorithm performs best for our data. We apply this algorithm systematically on the 24 estimated networks to detect communities of chronic diseases, i.e., multimorbidity patterns, for each sex and each age group at specific time-point.

**Fig. 1:**
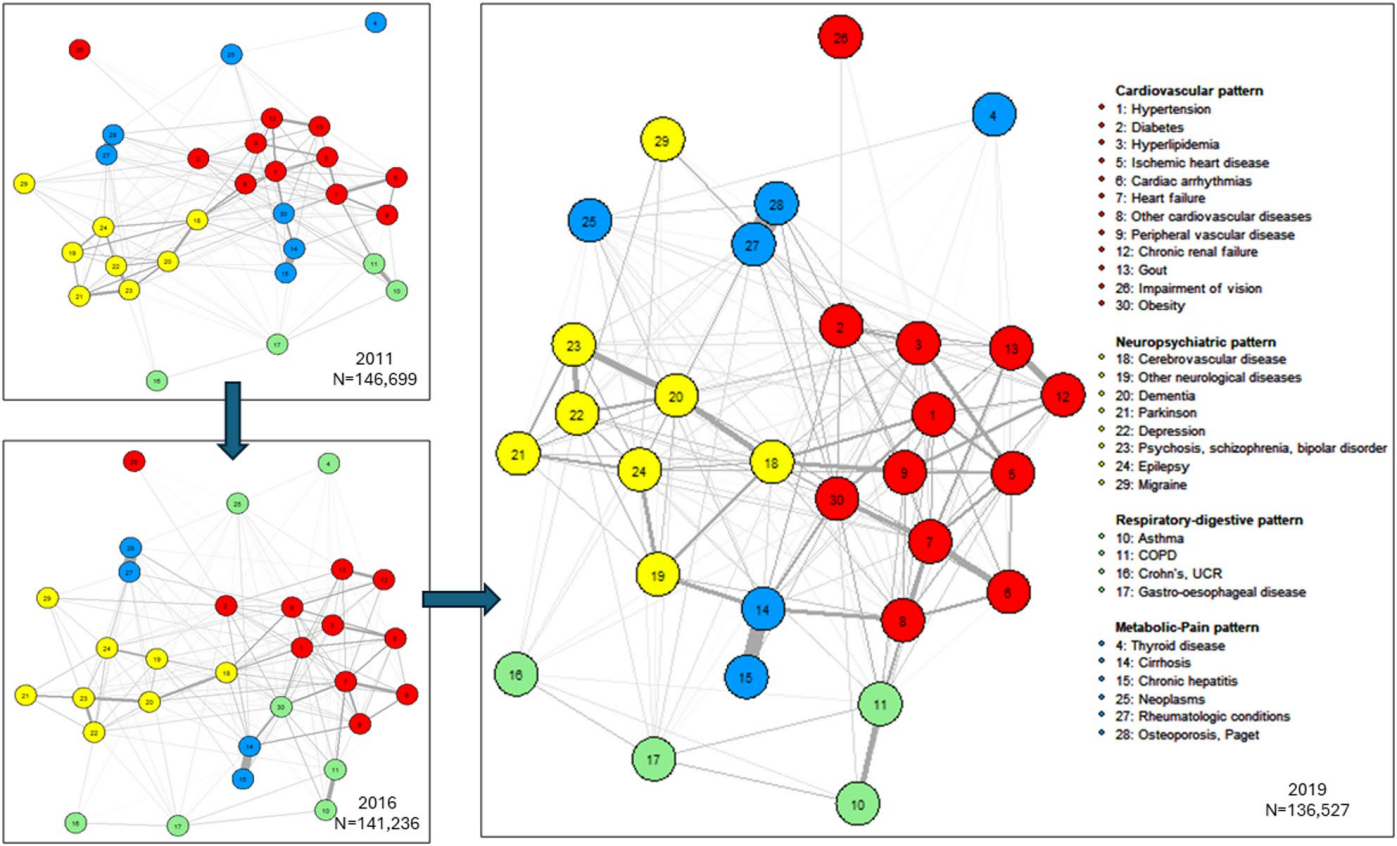
Estimated network of chronic conditions and corresponding multimorbidity patterns, females aged 60–69 at census, evaluated in 2011, 2016, 2019

As illustration, the Fig. 1 below displays the estimated network of chronic conditions and the corresponding multimorbidity patterns for females aged 60–69 at census, evaluated in 2011, 2016 and 2019. The number of survivors whose chronic diseases information are used to estimate each network is also reported. The estimated networks for other sub-populations are reported in the supplementary files. Across time, we identified four coherent groups of chronic conditions using the optimal algorithm. For the ease of interpretation, and also to be comparable with previous research done on similar population of Emilia-Romagna region by [14], we label each group of diseases, resulting in the cardiovascular group (in red), the neuropsychiatric group (in yellow), the respiratory-digestive group (in green), and the metabolic-pain group (in blue).

Within each coherent group identified, two categories of diseases can be recognized. The first category contains the main diseases that characterize the pattern (e.g., heart failure and cardiac arrhythmias in cardiovascular pattern, dementia and epilepsy in neuropsychiatric pattern), and the second category contains the conditions related to the main diseases (e.g., hypertension in cardiovascular pattern, thyroid diseases in metabolic-pain pattern). For all sexes and age groups, the composition of the cardiovascular pattern and the neuropsychiatric pattern remains almost identical across time. The composition of the respiratory-digestive and metabolic-pain patterns is more subject to change, but the main diseases that characterize these patterns are consistent. The detailed pattern composition for each sub-group and each time-point can be found in the supplementary files. The identified patterns at highest ages (i.e., for patients aged 80 and above at 2011 census and for patients aged 70–79 at census and ended up aged older than 80 by the end of observation period in 2019) are also more susceptible to variation. For the same age cohort, the multimorbidity patterns of female patients are more consistent across time than that of males patients.

Due to the chronicity of the diseases in this study, once affected, these diseases remain in the network until the individuals diagnosed with these diseases are selected out of the population. Thus, since the network of chronic disease is estimated from the information provided by the survivors at each time-point, each network is governed by two selection effects. The first selection acts through age (i.e., biological aging unrelated to morbidness), and the second selection acts through the fatality of the chronic conditions that patients are affected with (i.e., the complications of morbidness). But for all identified multimorbidity patterns, we observe that while the main diseases that characterize the multimorbidity pattern stay relatively stable, the related conditions to these main diseases might vary (e.g., obesity was classified in the metabolic-pain pattern in 2011 and became part of the cardiovascular pattern for females aged 60–69 at census and alive in 2019). Hence, even if the structure of the estimated chronic diseases network would necessarily change across time as the information obtained from the survivors to estimate these networks change at each time-point, which is caused by the change in number of survivors and the accumulation of chronic conditions among survivors as previously explained, the empirically-identified multimorbidity patterns structure remains stable.

**Fig. 2:**
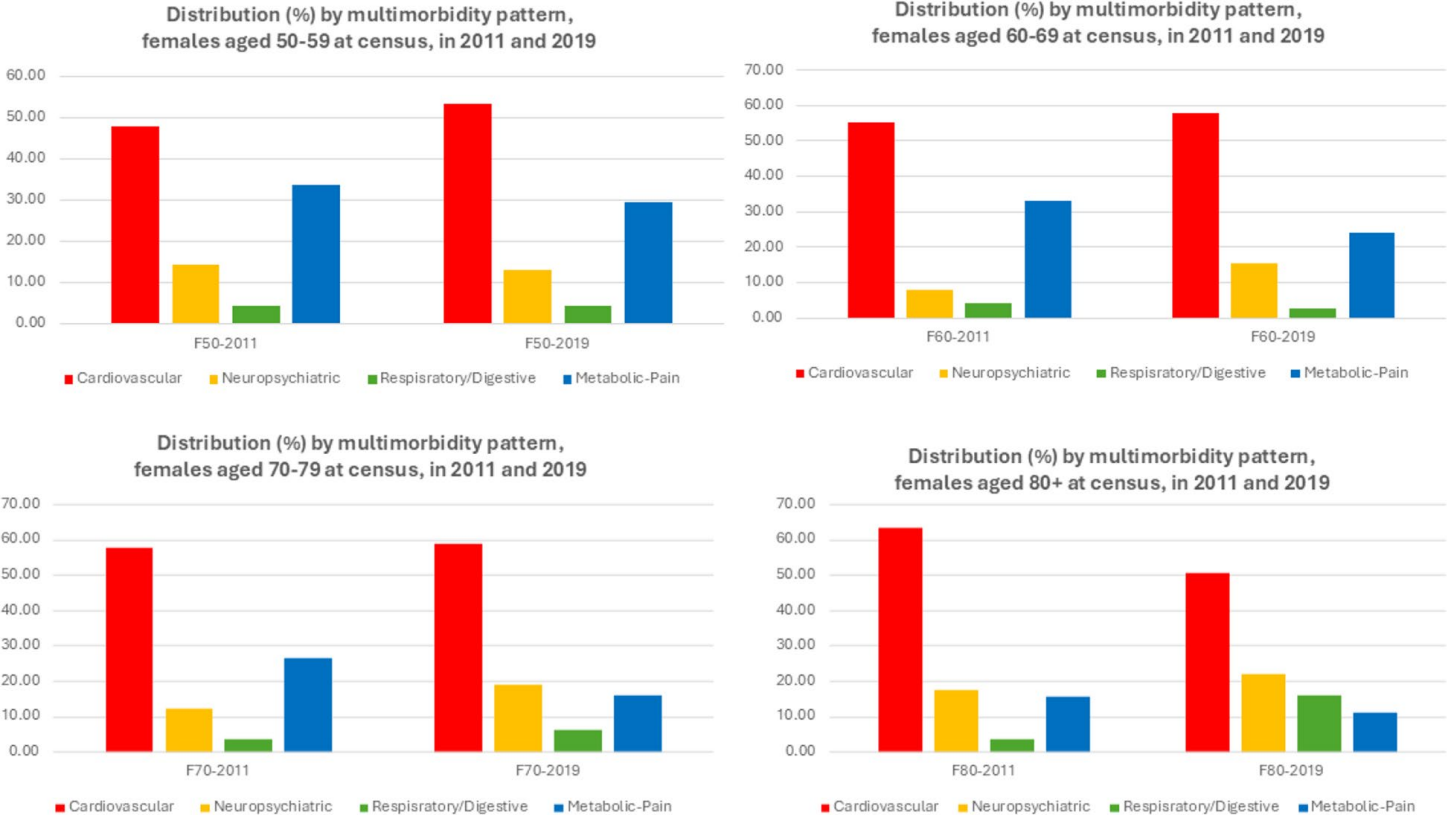
Distribution of multimorbidity patterns, female populations, aged 50–69, 60–69, 70–79 and 80 + at 2011 census, in 2011 and 2019

**Fig. 3:**
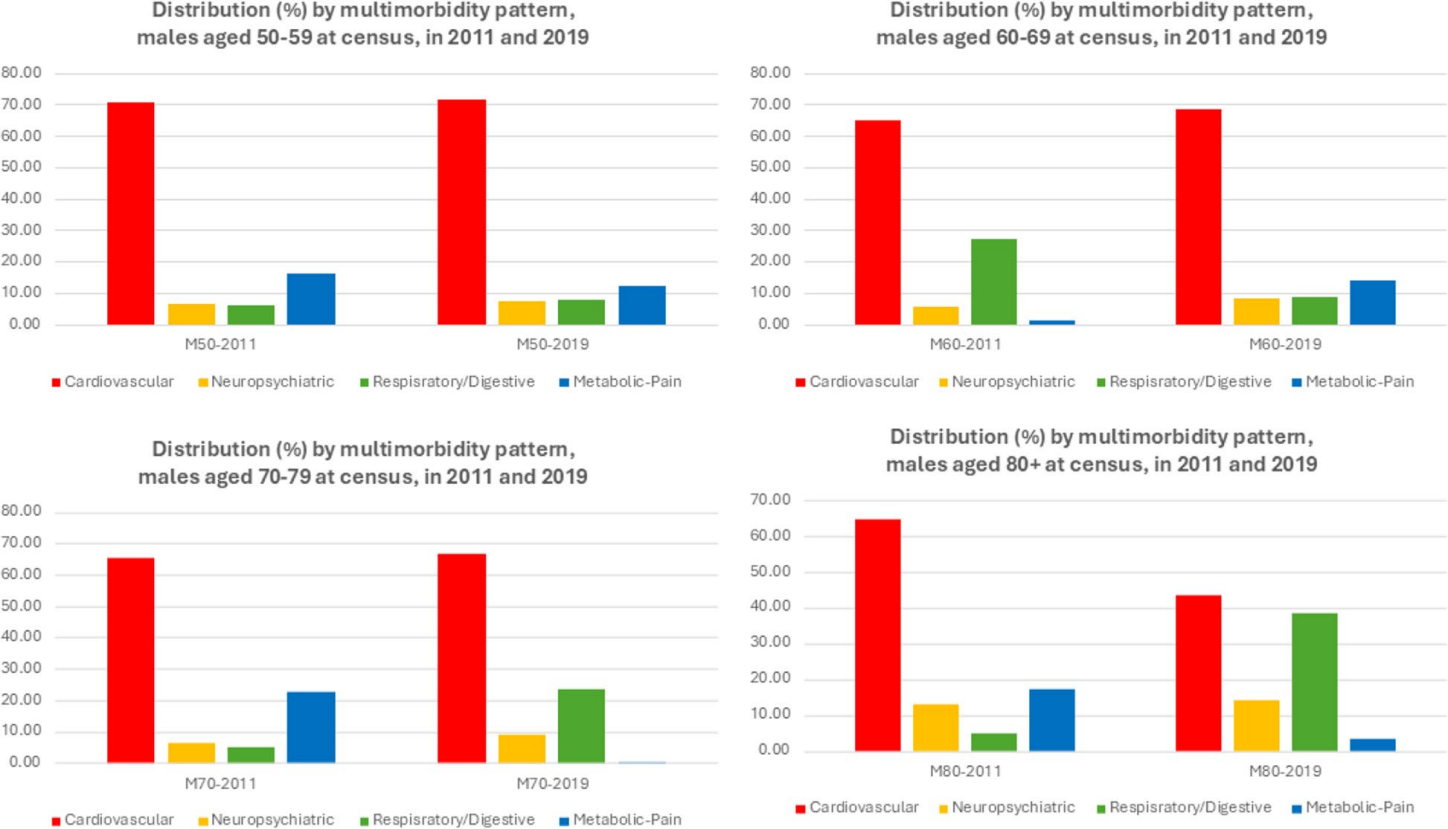
Distribution of multimorbidity patterns, male populations, aged 50–69, 60–69, 70–79 and 80 + at 2011 census, in 2011 and 2019

When the multimorbidity patterns structure is determined, we can also have an idea of the distribution of these patterns for each population in consideration, as represented in the Fig. 2 for females and Fig. 3 for males, at the beginning and at the end of the observation period. For females, the cardiovascular pattern is the most prevalent for all age cohorts at both time-points, followed mostly by the metabolic/pain pattern at the beginning and more variations by the end of the period observation. Similar observations can be drawn for male populations. It is noteworthy however that these distributions reflect the multimorbidity situation of different age cohorts that were separately identified from 2011 census data and should be studied separately. Not only there are cohort-specific characteristics that our data cannot allow for more detailed knowledge, but the selection effect by age and by fatality of the chronic diseases also has decisive impact on the multimorbidity patterns and the corresponding components that we can derive.

Moreover, obtaining clusters of chronic diseases without preconception, either by medical reasoning or other rationale, is also another objective of this study, as this practice could help bring to light unfamiliar co-occurrences of chronic diseases and potentially could point to novel direction of understanding multimorbidity conditions at older ages. The labels that we give to each group hold rather suggesting value and are meaningful enough to facilitate the discussion for the rest of this study and to be comparable with previous study done on the same population [14].

### Multimorbidity conditions and mortality at older ages

#### Impact of multimorbidity patterns on mortality of older multimorbid patients

In this section, two questions are of particular interests. We look to verify whether the newly determined multimorbidity patterns, though meaningful and consistent as previously documented, can improve our ability to assess mortality at older ages, and to what extent they can help explain mortality at older ages.

We first compare the performance of the model using multimorbidity patterns with traditional way of accounting for multimorbidity using the total number of chronic diseases that individuals accumulate across old ages to assess mortality at older ages. As described in the method section, a set of two Cox models is fit systematically to the 8 datasets of 8 sub-populations data (males and females stratified to 4 age-group cohorts). Both models control for the level of education and the rural/urban residence. The comparison of model’s performance is based on AIC, these values are reported in the Table 2 for each model fitted on each sub-population data. For males and females across all age-group cohorts, the model using only multimorbidity patterns variable performed consistently better than the model using only the total number of chronic diseases.

**Table 2:** Reported AIC values of each Cox model for each sub-population stratified by age group and sex from model using only total number of chronic diseases variable and only multimorbidity patterns variable

Sub-populations	Model using number of chronic diseases	Model using multimorbidity patterns
F50-59	128,612.80	121,264.00
F60-69	331,023.90	313,751.00
F70-79	977,667.50	927,749.30
F80 +	2,372,692.00	2,240,312.00
M50-59	189,255.00	178,416.40
M60-69	511,785.50	484,454.70
M70-79	1,183,393.00	1,119,712.00
M80 +	1,411,707.00	1,333,356.00

However, the better performance of the newly established multimorbidity patterns across all age groups and sexes does not imply that this variable should substitute the traditional approach in the literature. Notably, through the total number of chronic diseases, we have an indication of the severity of the multimorbidity condition of the individual. Meanwhile, through the multimorbidity patterns, we understand better the nature of the chronic diseases that the patients are suffering from. We run again systematically the Cox model with the total number of chronic diseases, the time-dependent variable of multimorbidity patterns across three time-points, the level of education and the urbanity of residence place to assess the impact of multimorbidity on different health outcomes. The results are reported in the Table 3 below.

**Table 3:** Cox model hazard ratios (95% CI) of the multimorbidity patterns in mortality of older individuals, females and males aged 50–59, 60–69, 70–79 and 80 + at 2011 census

	Females				Males			
	F50-59	F60-69	F70-79	F80 +	M50-59	M60-69	M70-79	M80 +
Multimorbidity patterns								
Cardiovascular pattern (ref.)	-	-	-	-	-	-	-	-
Neuropsychiatric pattern	1.46*** (1.36–1.57)	1.27*** (1.21–1.32)	1.32*** (1.29–1.35)	1.44*** (1.42–1.46)	1.83*** (1.71–1.95)	1.62*** (1.55–1.69)	1.61*** (1.57–1.65)	1.48*** (1.45–1.51)
Respiratory/Digestive pattern	1.17** (1.05–1.30)	1.27*** (1.17–1.37)	0.71*** (0.67–0.76)	0.88*** (0.85–0.91)	1.57*** (1.46–1.68)	2.08*** (2.00–2.17)	0.79*** (0.77–0.82)	0.83*** (0.81–0.86)
Metabolic/Pain pattern	1.07* (1.00–1.14)	1.13*** (1.08–1.18)	1.12*** (1.09–1.15)	0.67*** (0.66–0.68)	1.59*** (1.49–1.69)	0.96 (0.91–1.00)	2.57*** (2.49–2.65)	0.80*** (0.78–0.82)
Number of diseases	1.23*** (1.22–1.25)	1.22*** (1.22–1.23)	1.17*** (1.17–1.17)	1.04*** (1.04–1.04)	1.23*** (1.22–1.24)	1.20*** (1.20–1.21)	1.15*** (1.15–1.15)	1.04*** (1.04–1.04)

The Cox model for each age-group cohort also controlled for socioeconomic characteristics including the education level and the urbanity of residence place. These results are reported in Table 4. The full model’s results for each subgroup can be found in the appendix A.2

^***^p < 0.001

^**^p < 0.01

^*^p < 0.05, standard errors given in parentheses

**Table 4:** Cox model hazard ratios (95% CI) of education and urbanity in mortality of multimorbid individuals aged 50 and above, with time-dependent multimorbidity patterns and number of chronic diseases

	Females				Males			
	F50-59	F60-69	F70-79	F80+	M50-59	M60-69	M70-79	M80+
Education								
No education/primary (ref.)	-	-	-	-	-	-	-	-
Lower second education	0.91**(0.84-0.97)	0.94**(0.91-0.97)	0.92***(0.90-0.95)	0.88***(0.86-0.90)	0.78***(0.74-0.83)	0.85***(0.83-0.88)	0.86***(0.84-0.87)	0.91***(0.89-0.93)
Upper second education	0.89**(0.82-0.96)	1.00(0.95-1.06)	0.92***(0.88-0.96)	0.87***(0.85-0.90)	0.65***(0.61-0.69)	0.81***(0.78-0.84)	0.82***(0.80-0.85)	0.88***(0.85-0.90)
University	0.81***(0.73-0.90)	0.85***(0.79-0.91)	1.01(0.95-1.08)	0.87***(0.83-0.91)	0.58***(0.53-0.64)	0.68***(0.64-0.72)	0.75***(0.72-0.79)	0.91***(0.88-0.95)
Urbanity of residence								
Cities (ref.)	-	-	-	-	-	-	-	-
Small cities/Suburban	0.86***(0.81-0.91)	0.94**(0.90-0.97)	1.05***(1.02-1.07)	1.02*(1.00-1.03)	0.91***(0.87-0.96)	0.96**(0.93-0.99)	1.01(0.99-1.03)	1.00(0.98 - 1.02)
Rural	0.93*(0.86-1.00)	1.05(1.00-1.10)	1.15***(1.12-1.18)	1.09***(1.08-1.11)	1.08**(1.02-1.14)	1.05**(1.01-1.09)	1.12***(1.09-1.15)	1.08***(1.06-1.10)

The impacts on mortality of multimorbidity patterns and number of chronic diseases are reported in table 3. The full model’s results for each subgroup can be found in the appendix A.2.

***p < 0.001

**p < 0.01

*p < 0.05, standard errors given in parentheses

We found that the variables accounting for multimorbidity conditions of older patients, including the multimorbidity patterns and the number of chronic diseases, statistically significantly increase the hazard rates of dying across all sexes and age groups with only few exceptions. The impact of the number of diseases steadily aggravates mortality for both males and females, at younger old ages as well as at oldest ages. As for the impact of multimorbidity patterns, compared to individuals having cardiovascular pattern, individuals having any other pattern always experience higher mortality, except for the respiratory/digestive pattern at older ages for females and males and for the metabolic/pain pattern at highest ages. In particular, individuals with neuropsychiatric pattern encounter consistently higher mortality across all sexes and age groups. This impact is particularly high at younger old ages (i.e., for females aged 50–59 at 2011 census, the risk is 1.46 [1.35, 1.57] times higher; for males aged 50–59 at 2011 census, the risk is 1.83 [1.71, 1.95] times higher), which strongly suggests a need for more attention to the neurological conditions and the mental health at this age range of both sexes. Meanwhile, compared to individuals with cardiovascular pattern, those with metabolic/pain pattern not only suffer from higher mortality but this impact remains stable throughout old ages until highest ages.

#### Impact of socioeconomic characteristics on mortality of older multimorbid patients

##### Education

We found statistically significant protective effect of education on mortality of older patients with chronic diseases multimorbidity. Compared to having no education or only primary education, higher levels of education display consistent positive effects in reducing the risk of death in all age groups for females and males. Of which, the protective impact of having an university degree compared to having no education or only primary education appeared to be stronger for males than for females at younger old ages. Females aged 50–59 at census with an university degree has 0.81 [0.73, 0.90] times lower risk of decease than those with no or primary education, meanwhile this ratio is 0.58 [0.53, 0.64] times for males. At ages 60–69, the corresponding estimation is 0.85 [0.79,0.92] for females and 0.68 [0.64, 0.84] for males.

At oldest ages (i.e., 80 and above), the impact of having an university degree compared to the lower education level sharply decreases for males, at 0.91 [0.88, 0.95] compared to 0.87 [0.84, 0.91] in females. From previous study, it is observed that females continue to display advantage in mortality compared to their male counterparts [21]. Narrowing to males with multimorbidity who survived until age 80 and above, our results indicate that higher education holds important role in male survivorship. In one’s lifetime, education often ends at the early part of adulthood and becomes correlated with employment, health literacy and income, which in turns can have an impact on longevity, especially for the oldest cohort where university education was selective and reflects past economic conditions of the individuals. Our data is not enough to investigate this path, but as observed from Table 1, even though only a small proportion of oldest patients with co-morbidity identified at 2011 census achieved university degree (2.13% for females and 4.92% for males), we can still gauge a significant protective effect of a university degree on mortality. Our results hence strongly suggest that education is beneficial for the survival of multimorbid patients at older ages, and this impact remains consistently non-negligible until highest ages for both males and females.

##### Urbanity of the residence place

For both females and males, living in suburban is statistically significantly associated with lower risk of dying at younger old ages (i.e., aged 50–59 and 60–69 at 2011 census) compared to those living in urban areas. This trend is inverted at older old ages (i.e., aged 70–79 and 80 + at 2011 census) for females, though no statistically significant impact is found for older males. When living in rural areas is considered, males living in rural areas are found to be associated with higher mortality than males living in urban areas for all older age-cohorts.

For females, living in rural areas continues to be associated with a statistically significant protective effect at youngest old ages (i.e., aged 50–59 at census). Starting age 70 and until highest ages, living in rural is associated with higher risk of mortality compared to those living in urban areas. This change in the direction of the observed impacts suggests that as ages increase, the multimorbidity conditions of both females and males might require more intensive medical interventions, and limited access to healthcare of good quality in rural areas is taxing for mortality of multimorbid patients at older ages. This observation highlights the need for further investigation into disparities in healthcare access and associated social inequalities among older people living in urban versus rural areas.

###  Impact of the gatekeepers diseases on health outcomes at older ages

**Fig. 4:**
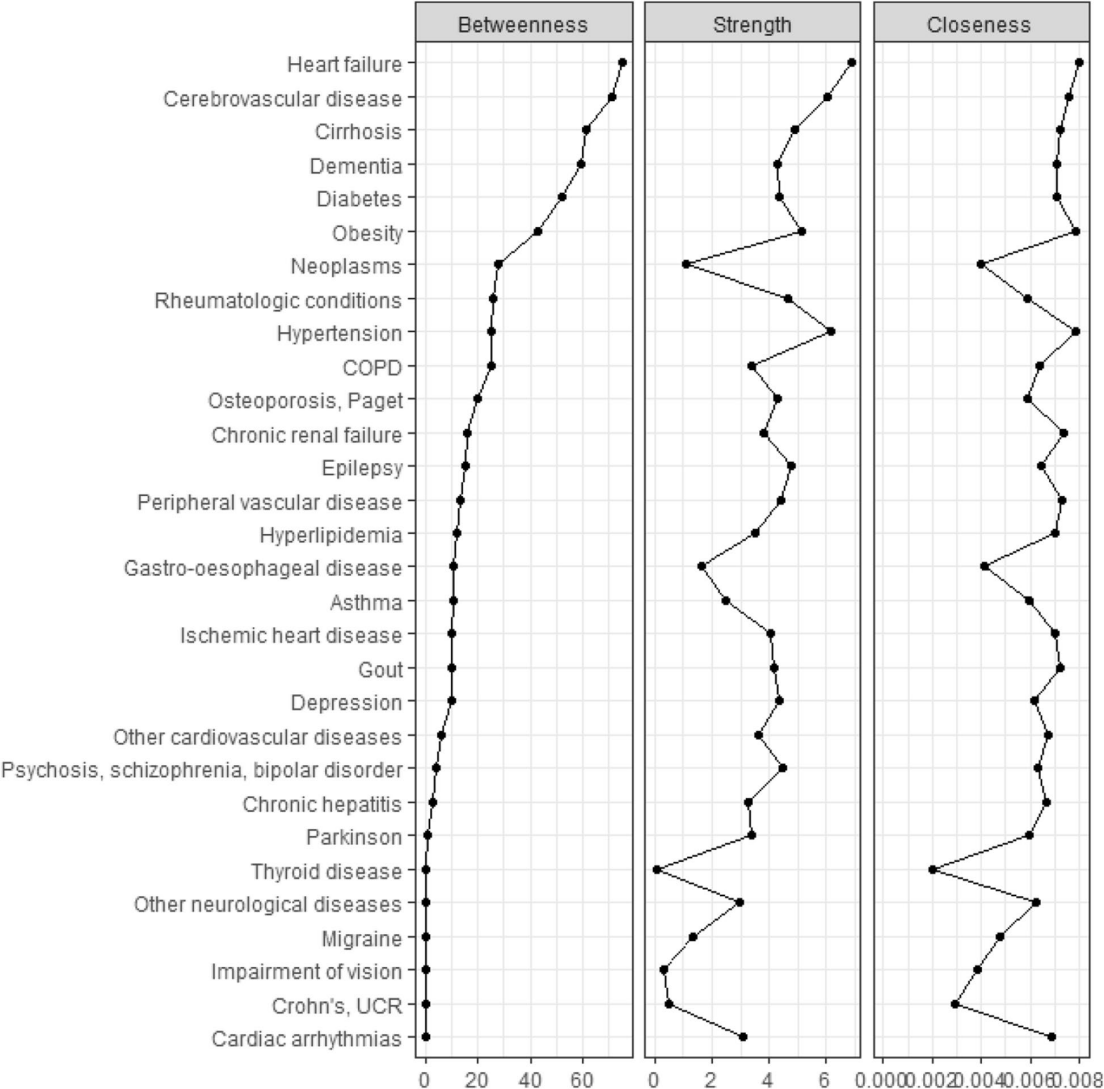
Centrality measures, females aged 60–69 at census 2011, followed up to 2019

The advantage of studying chronic diseases under the network perspective is not limited to the establishment of multimorbidity patterns. Using the centrality measures (i.e., betweenness centrality, strength centrality and closeness centrality), we assessed the prominence of each node disease. A total of 24 networks was estimated (for 2 sexes, 4 age groups and at 3 times points in 2011, 2016 and 2019). For each estimated network, centrality measures are produced, as illustrated in the Fig. 4 below, and can be provided upon requests.

Among these three metrics, we focus on the betweenness centrality. This metric measures the extent to which a disease stays on the shortest path connecting two other diseases in the network, i.e., how many shortest paths linking two given diseases have to pass through this specific disease. We term these diseases the ”gatekeeper diseases”. Across sexes and age-group cohorts, we found that the diseases with the highest rank in terms of betweenness centrality oftentimes also rank high in strength centrality (i.e., they are strongly correlated with other diseases), and in closeness centrality (i.e., they locate close to other diseases within the estimated network), especially for the top five diseases with the highest betweenness centrality. It is noteworthy that the gatekeepers diseases are not necessarily always the diseases with highest prevalence.

For example, the top five gatekeepers diseases for females aged 60–69 at census in 2011 reported in the Fig. 4 are heart failure, cerebrovascular disease, cirrhosis, dementia and diabetes, with respective prevalence of 1.09%, 7.51%, 1.90%, 3.29% and 20%. Even if the regression model used in this study cannot allow for causality inference, our results still suggest that given the consistent bridging position of the top gatekeepers diseases within the network, there is possibility that focusing on them can contribute to break the paths connecting chronic conditions, reduce the formation of multimorbidity patterns for the population in consideration. The gatekeepers diseases can act as potential target for public health intervention.

**Fig. 5:**
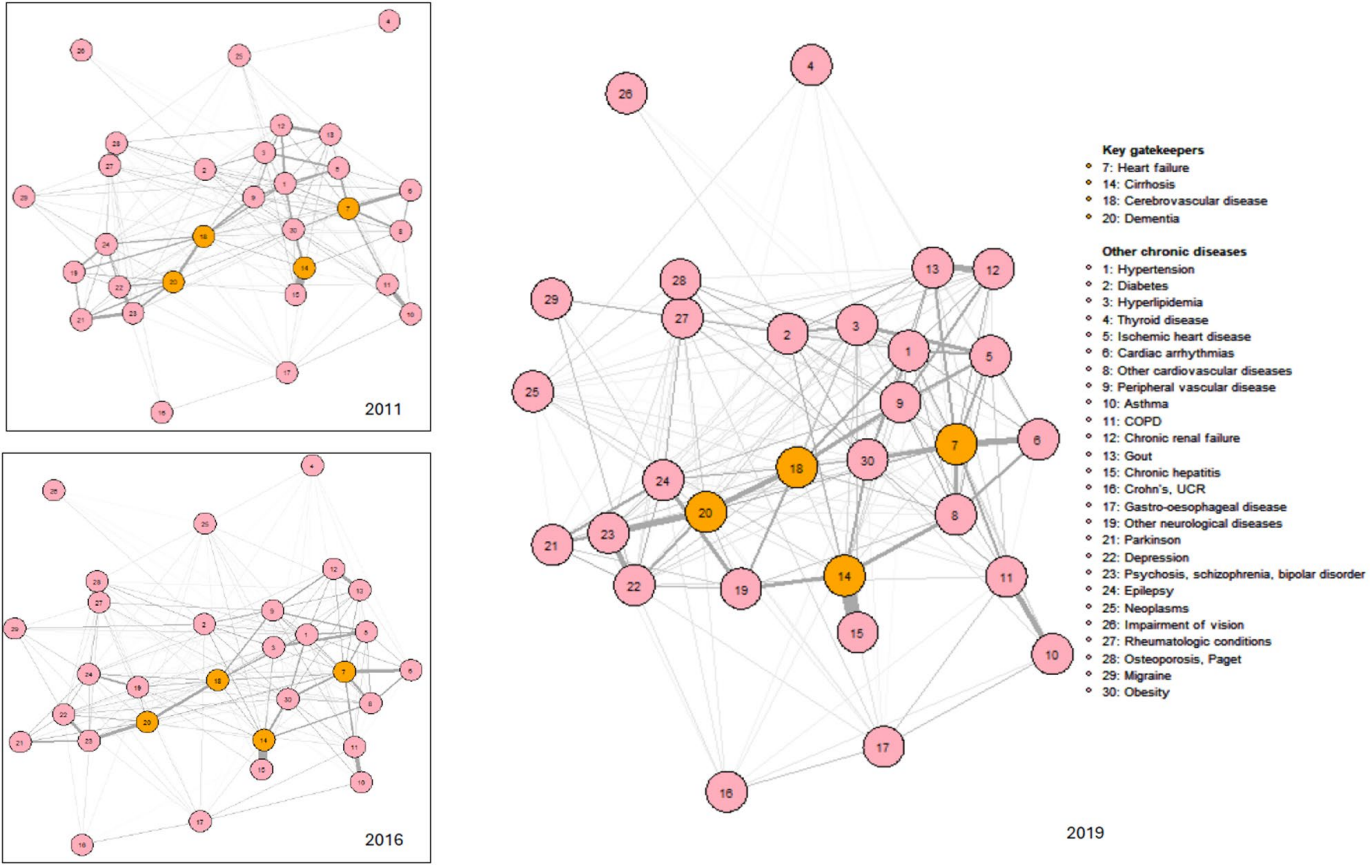
Gatekeepers diseases (the chronic conditions with the highest betweenness centrality measures), females aged 60–69 at census 2011 and followed up to 2019

Another reason to make the identification of the top gatekeepers diseases worthwhile is the consistency of their position. As illustrated in the Fig. 5 below, even when the network structure would necessarily change at each time point as individuals passed away across observation period, 4 gatekeeper diseases out of top five previously mentioned appear consistently at each time-point for the cohort of female sexagenarians. Thus, these top gatekeepers diseases are not fatal conditions, but they are persistent among survivors and play an important role in not only maintaining the chronic diseases network but also giving way to the onset of other chronic diseases. This result is consistently observed for all sexes and age-group cohorts.

**Fig. 6:**
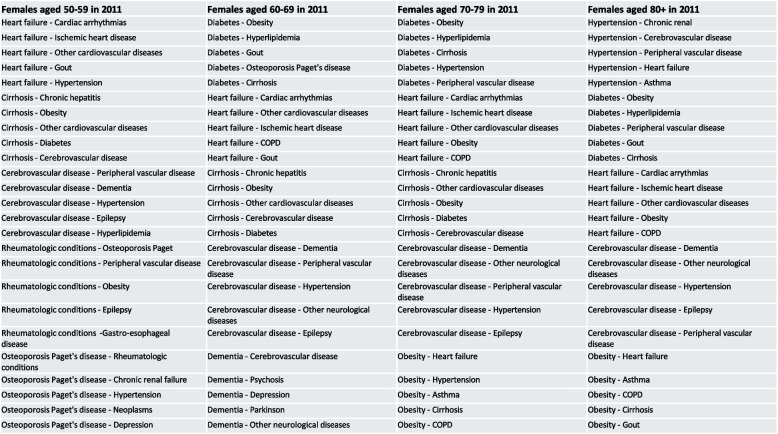
Strongest dyads with top five gatekeepers diseases for females of each age cohort in 2011

**Fig. 7:**
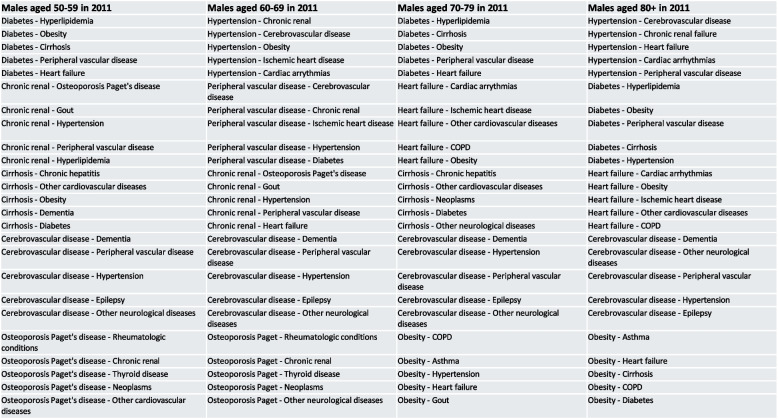
Strongest dyads with top five gatekeepers diseases for males of each age cohort in 2011

We also investigate the impact of early detection of these key gatekeepers in 2011 on health outcomes observed across the observation period, including the number of unplanned hospitalization, the length of stay in hospital and the mortality. For each subgroup, we sort the dyads with each top gatekeeper disease identified in 2011 by strength of association, and select the top five dyads that hold the strongest link for each gatekeeper, i.e., we identify the 25 strongest multimorbid dyads that directly involve the top gatekeepers diseases. Then we classify each subpopulation into two groups of patients: those who have at least one of these 25 strongest dyads and those who do not, and fit a set of three different models corresponding to each health outcome to each group, control for the level of education and the urban/rural place of residence. The list of these 25 strongest dyads with top gatekeepers is given in the Figs. 6 and 7 below, and the results obtained from the regression models are reported in the Tables 5 and 6 respectively for females and males.

**Table 5:** Impact (odds ratio, incidence rate ratio, multiplicative change) of having at least one of the strongest dyads with the top five gatekeepers diseases in 2011 on different health outcomes until 2019, females aged 50–59, 60–69, 70–79 and 80 + at 2011 census

	Females			
	F50-59	F60-69	F70-79	F80 +
Top five gatekeepers in 2011	Heart failure Osteoporosis Paget’s disease Cerebrovascular diseases Rheumatologic conditions Cirrhosis	Heart failure Dementia Cirrhosis Diabetes Cerebrovascular diseases	Heart failure Cerebrovascular diseases Diabetes Obesity Cirrhosis	Diabetes Cerebrovascular diseases Heart failure Hypertension Obesity
Mortality 2011–2019	1.51*** (1.41–1.62)	2.73*** (2.61–2.86)	2.45*** (2.38–2.52)	2.83*** (2.75–2.92)
Unplanned hospitalization 2011–2019	1.26*** (1.23–1.29)	1.65*** (1.61–1.69)	1.64*** (1.62–1.67)	1.40*** (1.39–1.42)
Length of stay 2011–2019	1.24** (1.08–1.43)	7.38*** (6.44–8.46)	10.41*** (9.43–11.51)	5.07*** (4.69–5.48)

The list of the five strongest dyads with each of the five gatekeepers (i.e., the 25 strongest dyads) for each male age-group cohort in 2011 is given in Fig. 6. The model also controlled for education level and urbanity of the residence place. These results can be found in the supplementary files

^***^p < 0.001

^**^p < 0.01

^*^p < 0.05, standard errors given in parentheses

**Table 6:** Impact (odds ratio, incidence rate ratio, multiplicative change) of having at least one of the strongest dyads with the top five gatekeepers diseases in 2011 on different health outcomes until 2019, males aged 50–59, 60–69, 70–79 and 80 + at 2011 census

	Males			
	M50-59	M60-69	M70-79	M80 +
Top five gatekeepers in 2011	Diabetes Cirrhosis Cerebrovascular diseases Chronic renal failure Rheumatologic conditions	Cerebrovascular diseases Chronic renal failure Peripheral vascular disease Osteoporosis Paget’s disease Hypertension	Heart failure Cerebrovascular diseases Diabetes Obesity Cirrhosis	Diabetes Cerebrovascular diseases Heart failure Hypertension Obesity
Mortality 2011–2019	2.20*** (2.08–2.34)	2.44*** (2.39–2.53)	2.38*** (2.32–2.45)	2.46*** (2.36–2.56)
Unplanned hospitalization 2011–2019	1.52*** (1.48–1.57)	1.77*** (1.74–1.81)	1.50*** (1.48–1.52)	1.34*** (1.32–1.36)
Length of stay 2011–2019	2.33*** (1.99–2.74)	14.37*** (12.66–16.31)	7.02*** (6.39–7.70)	3.50*** (3.23–3.80)

The list of the five strongest dyads with each of the five gatekeepers (i.e., the 25 strongest dyads) for each male age-group cohort in 2011 is given in Fig. 7. The model also controlled for education level and urbanity of the residence place. These results can be found in the supplementary files

^***^p < 0.001

^**^p < 0.01

^*^p < 0.05, standard errors given in parentheses

#### Gatekeeper diseases and mortality at older ages

The results in Table 5 suggest that females having multimorbidity involving at least one of the strongest dyads with the top five gatekeepers diseases at the beginning of the observation period experience consistently higher risk of death across all age-group cohorts, ranging from 1.51 [1.41, 1.62] times higher risk at youngest old ages to 2.83 [2.75, 2.92] times higher risk at oldest ages. Similar remarks can be drawn from male results as reported in Table 6, though the increasing trend is not as striking, ranging from 2.20 [2.08, 2.34] times higher risk at youngest old ages to 2.46 [2.36, 2.56] times higher risk at oldest ages.

#### Gatekeeper diseases and unplanned hospitalization

Having multimorbid condition with the top gatekeepers diseases is found to consistently increase the rate of being hospitalized significantly for both females and males across all age-group cohorts. For females, the highest increase in rate of unplanned hospitalization following having multimorbid with top gatekeeper diseases compared to those who did not is observed for cohorts aged 60–69 and 70–79 at 2011 census, with estimations at 1.65 [1.61, 1.69] and 1.64 [1.62, 1.67] times higher respectively. Both groups, though undoubtedly have cohort-specific differences, are found with similarities in the top gatekeepers diseases that they were diagnosed with, including heart failure, cerebrovascular diseases, diabetes, and cirrhosis. This result reaffirms the consistency of the gatekeepers diseases in characterizing the multimorbidity conditions at older ages (not only within a given age-group cohort across time, but also across age-group cohorts), and the impacts that these diseases have on health outcomes of older patients.

For males, being diagnosed with multimorbidity involving top gatekeepers is associated consistently with higher rate of unplanned hospitalization, with the highest estimation at 1.77 [1.74, 1.81] times higher found for cohorts aged 60–69 at 2011 census. It is noteworthy to recall that from Table 4, living in rural is statistically significantly associated with higher risk of death of male patients aged 60–69, for which we previously hypothesized that one of the reasons might be due to the need of medical intervention that healthcare facilities in rural areas might not be sufficient. Herein, this hypothesis is enhanced with the observed increase in unplanned hospitalization of males aged 60–69 at census due to being affected with gatekeepers diseases identified for the age groups (e.g., cerebrovascular diseases, chronic renal failure, peripheral vascular diseases, osteoporosis and Paget’s diease, and hypertension).

#### Gatekeeper diseases and length of hospital stay

For females and males, we continue to observe persistent trend where having multimorbidity with top gatekeepers diseases increase the length of being hospitalized. In particular, for male cohorts aged 60–69 at census, the length of stay in hospital also increases by a factor of 14.37 [12.66, 16.31] compared to males in the same cohorts but affected with multimorbidity that did not involve the identified gatekeepers diseases. For the specific case of Emilia-Romagna region, this result gave strong signal that more attention should be given to this sub-group of male patients (i.e., those aged 60–69 at census and affected with the detailed top five gatekeepers diseases). Similar results were also found for female population, with the highest increase in length of stay in hospital was observed in females aged 70–79 at census.

**Fig. 8:**
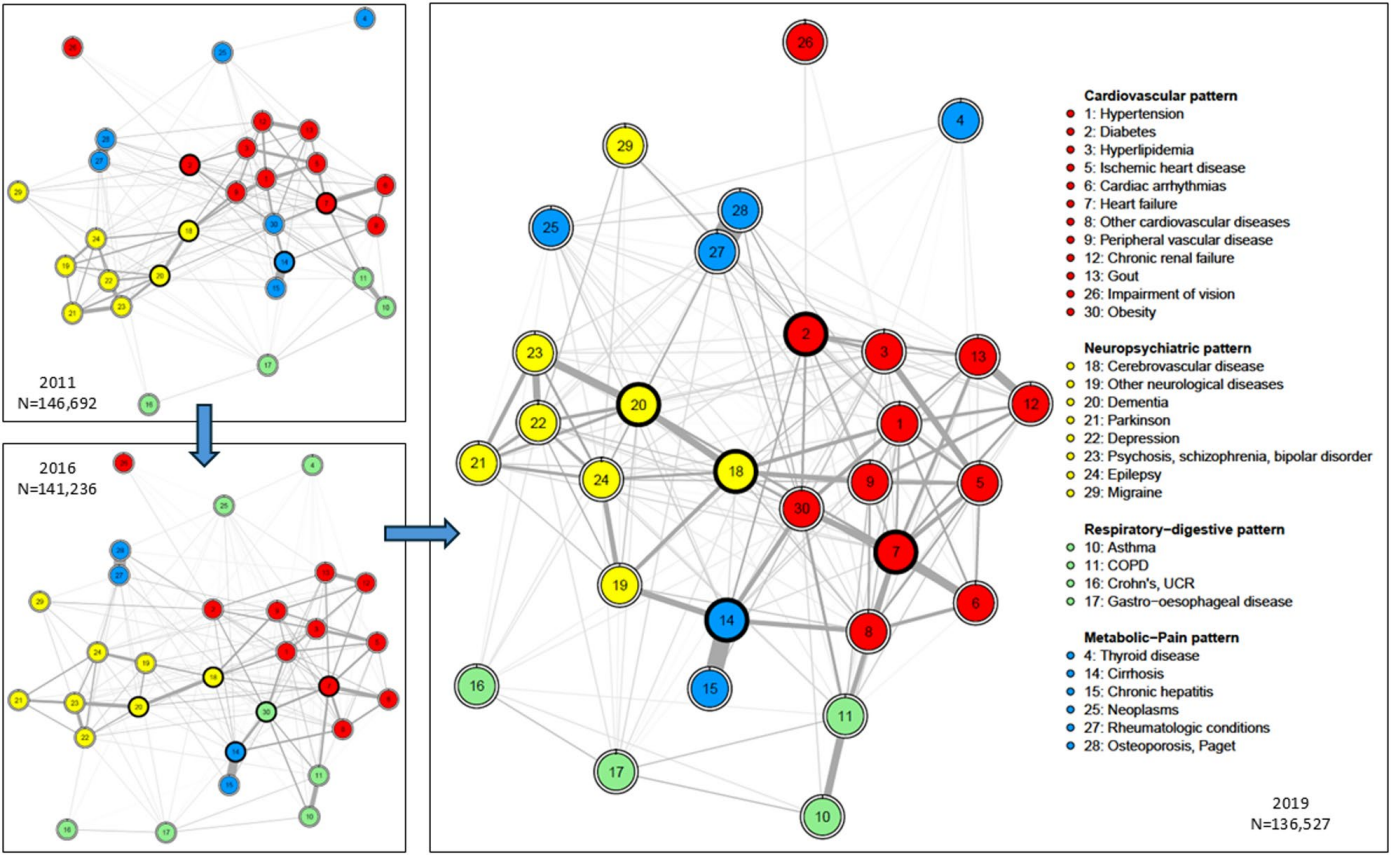
Estimated network of chronic diseases with multimorbidity patterns and gatekeepers diseases (in bold black ring), females aged 60–69 at 2011 census, in 2011, 2016 and 2019

The Fig. 8 illustrates how we can visually integrate the top five gatekeepers diseases (i.e., diseases with highest betweenness centrality measures) to the network of multimorbidity conditions for monitoring purpose. We represent these gatekeepers diseases as nodes marked with black bold border while the multimorbidity patterns are color-coded and the association strength between pair of chronic diseases is visualized by the thickness of the edges. For females aged 60–69 at census, we found that across 8 years of observation, the top gatekeepers concentrate mostly in the cardiovascular and neuropsychiatric patterns. Combining with previous finding from Table 3 that the neuropsychiatric pattern is also the pattern with highest mortality, the identification of the top gatekeepers diseases within this pattern provides further argumentation for the choice of specific targets for intervention for each sex and each age group. At older ages, multimorbidity with chronic diseases is not only complicated in terms of treatment but also constitutes major financial and emotional burden for the patients and the society. Hence, each piece of evidence that can offer a chain of justification is valuable for timely intervention and efficient planning. If we were following the traditional approach based on the prevalence, it is likely that we overlooked important targets for public health policies.

## Discussion

In this paper, we applied and extended mixed graphical model and network analysis [12] to study the multi-morbidity patterns across time at older ages. Compared to earlier investigations, we extended our population to younger old ages (i.e., starting from age 50), and identified exhaustively the multimorbid older patients of one region (i.e., Emilia-Romagna region) rather than using survey data or relying on one-year observation. We also followed our cohorts from 2011 to 2019. Our analyses bring support to this approach as monitoring tool of the multimorbidity situation of a population at any given time, and illustrate an application of using network metric to extract insight for public health intervention.

Compared to traditional approach to network analysis of multimorbidity, graphical model provides a parsimonious way to integrate a large number of variables into one cohesive setting, facilitate regression models without jeopardizing by a large number of variables even with small data size, and avoid potential contradicting effects of different single diseases on health outcomes. Moreover, going beyond the use of network metrics such as centrality measures as purely descriptive tool, we identified potential diseases for public health intervention using these metrics, and investigated the role that the multimorbidity involving these target diseases can have in health outcomes of older patients from longitudinal perspective.

Our analysis framework is designed in a way that seeks to avoid as much as possible the arbitrary choices in every step of the procedure nor any preconception about the co-occurrence of the chronic conditions. We systematically followed each age-group cohort (i.e., aged 50–59, 60–69, 70–79 and 80 + at census 2011) of both sexes, and estimated their corresponding multimorbidity network and patterns at different time-points (at 2011, 2016 and 2019). As such, we were able to provide systematic observations for each age-group cohort across time, offering a set of specific insights of multimorbidity situation at older ages for evidence-based policies.

The combination of mixed graphical models and network analysis offers powerful tools for longitudinal study that we only start exploring. Of which, the observed consistency of clusters of chronic diseases across sexes, age groups and time points merits consideration. There are reasons to expect that some degrees of stability in the multimorbidity patterns can be expected, as our study focuses on chronic diseases that do not remit and accumulate across time among survivors. However, to our best knowledge, we are the first to verify this hypothesis of stability on good-quality data without any prior constraints and report only the patterns that came from data. From our point of view, these data-driven findings can contribute to provide the scientific-based evidence that stakeholders (e.g., policy planners and practitioners) are lacking in order to use multimorbidity patterns in further application, especially regarding healthcare and resources allocation in a context that patient-centered approach is increasingly prioritized. Further investigations, notably on a longer period and different populations, will be necessary to assess this empirical stability and its potential generalization.

Although the identification of multimorbidity patients in our study was based on extensive health data sources, we first focused on death and health outcomes following hospitalization. It is possible that the hospitalized older adults might be affected with different severity in diseases progress than those who were not hospitalized, and staying in the hospitals also makes patients more prone to diseases accumulation due to complication and infection. Hence it will be valuable in the future to investigate the impact of the multimorbidity patterns as identified herein on more aspects of health at older ages outside hospital context.

## Conclusion

For each sex and age group, we identified four consistent multimorbidity patterns: the cardiovascular pattern, the neuropsychiatric pattern, the respiratory/digestive pattern and the metabolic-pain pattern for the ease of interpretation. Across subpopulations, we observed that the identified patterns in both female and male populations are relatively stable across time (in 2011, 2016, 2019). This empirical consistency of multimorbidity patterns constitutes the necessary condition to ensure a degree of comparability while looking at the longitudinal trajectory of multimorbidity patterns for each age cohort across time, which remains under-documented in existing literature. This possibility to draw clusters of chronic diseases from network perspective highlights the existence of latent factors (e.g., medical, biological or social) in driving the accumulation of chronic diseases during aging process at older ages.

We also brought evidence that accounting for multimorbidity using the patterns identified by mixed graphical model and network analysis not only improves the performance of the mortality model but also allows for more information to be drawn from the results. The neuropsychiatric pattern, though not the most prevalent, is the leading group behind mortality at older ages, and even from the earliest age group of 50–59 for both sexes, which warrants higher attention for these pathologies at youngest old ages. Meanwhile, the level of education consistently has protective effect even at highest ages for both females and males, and the urbanity of the residence has more varying effects depending on age-group cohort. Since the multimorbidity experiences are likely to differ between males and females at different ages, the flexibility of our approach is valuable in providing a finer understanding of the health burden in the population and a practical guide for evidence-based policies.

We translated the observed characteristics of the chronic diseases network into practical insights for policies planning by introducing the notion of ”gatekeepers diseases”, which is defined as the chronic diseases with the highest betweenness centrality for each age-group cohort. Going beyond the conventional prevalence-based approach, we investigated and provided the support that early detection of gatekeepers diseases can contribute to prevent the multimorbid patients from suffering from higher mortality, more unplanned hospitalization and longer length of stay in hospitals. The gatekeepers diseases are not necessarily the diseases with highest prevalence but our results strongly support their potential as targets for intervention, which would have been overlooked if we were to follow a prevalence-based approach.

Studying chronic diseases multimorbidity that was estimated by mixed graphical model offers an intuitive understanding of how chronic diseases interplay with each others, a flexibility to identify target diseases for appropriate prevention depending on the problem at hand, and a possibility to cluster diseases into meaningful multimorbidity patterns. We found these three qualities recommendable for this approach to become a monitoring tool of the multimorbidity situation across time as well as produce more reliable support for evidence-based policies, such as developing individual frailty index using administrative health data.

## Supplementary Information

Supplementary Material 1.

Supplementary Material 2.

## Data Availability

The data are not publicly available due to ethical restrictions.
