# Supplementary material for: Population-level analysis of chronic disease multimorbidity at older ages across time using mixed graphical models
Source: Arch Public Health. 2026 Mar 17;84:87. doi: 10.1186/s13690-026-01886-3 (PMC13107661; doi:10.1186/s13690-026-01886-3)
Supplement: Supplementary file 1 — Supplementary Material 1. [file 13690_2026_1886_MOESM1_ESM.pdf]

Supplementary files

Figure 1: Estimated network of chronic diseases with multimorbidity patterns and gatekeepers diseases (in bold black ring), females aged 50-59 at 2011 census, in 2011, 2016 and 2019

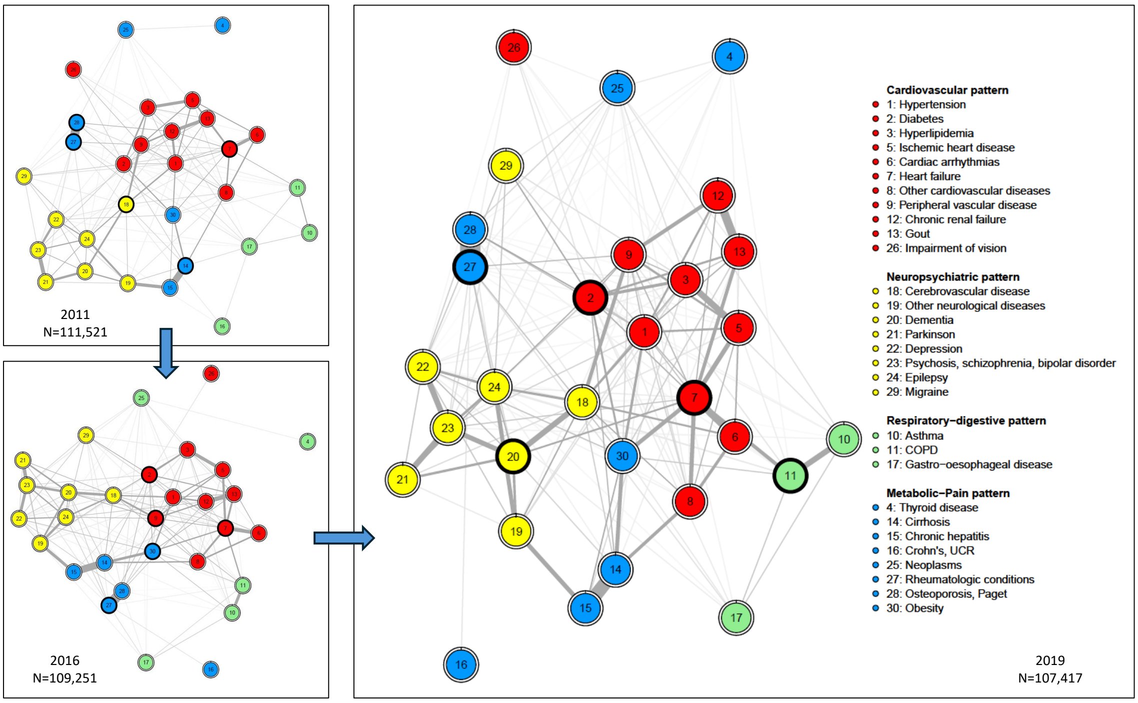

Notes: For the ease of interpretation and the comparability with results from previous research on similar population study by Fortuna et al. (2021), group 1 is termed cardiovascular patterns (in red), group 2 is termed is neuropsychiatric pattern (in yellow), group 3 is termed respiratory-digestive pattern (in green), group 4 is termed metabolic-pain pattern (in blue).

Figure 2: Estimated network of chronic diseases with multimorbidity patterns and gatekeepers diseases (in bold black ring), females aged 60-69 at 2011 census, in 2011, 2016 and 2019

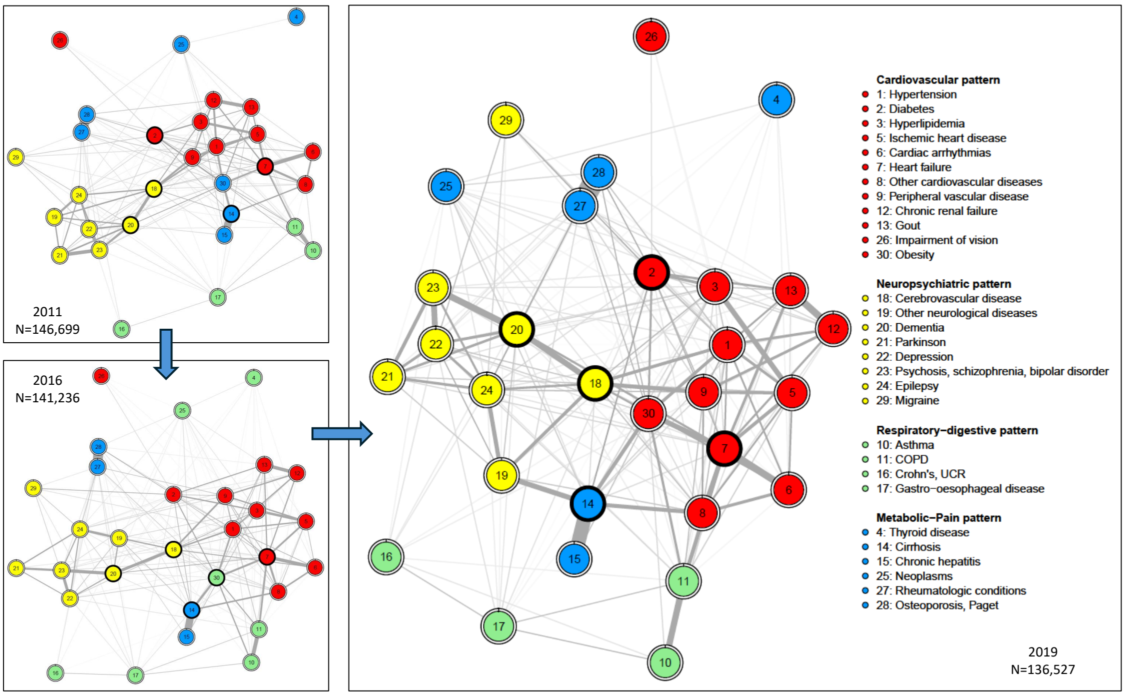

Notes: For the ease of interpretation and the comparability with results from previous research on similar population study by Fortuna et al. (2021), group 1 is termed cardiovascular patterns (in red), group 2 is termed is neuropsychiatric pattern (in yellow), group 3 is termed respiratory-digestive pattern (in green), group 4 is termed metabolic-pain pattern (in blue).

Figure 3: Estimated network of chronic diseases with multimorbidity patterns and gatekeepers diseases (in bold black ring), females aged 70-79 at 2011 census, in 2011, 2016 and 2019

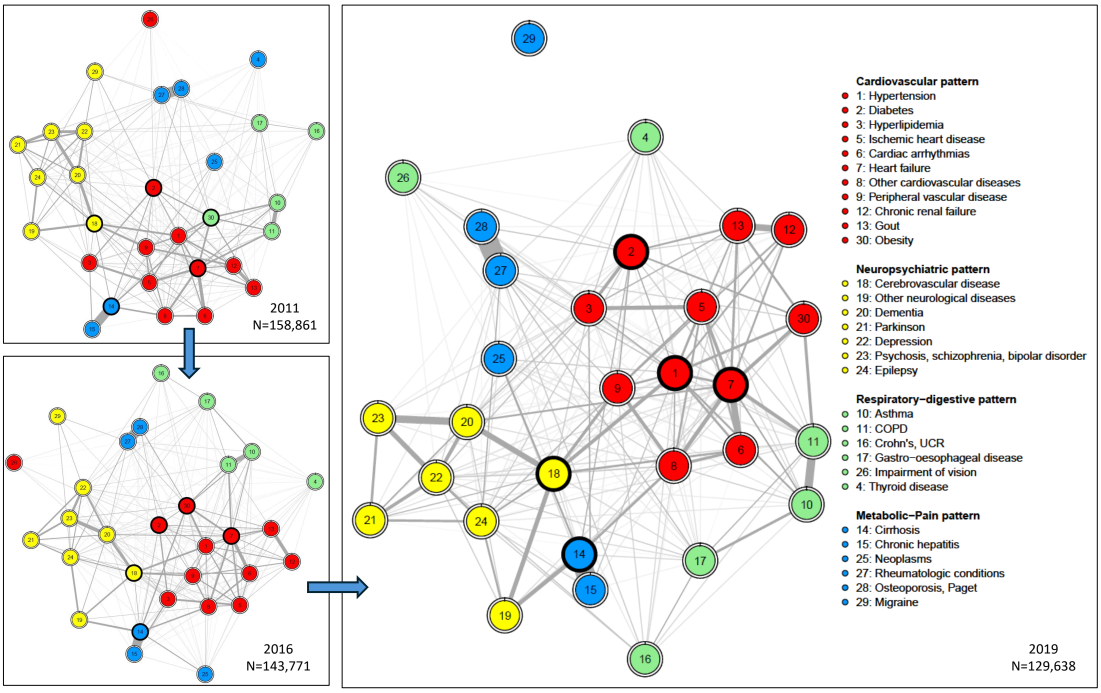

Notes: For the ease of interpretation and the comparability with results from previous research on similar population study by Fortuna et al. (2021), group 1 is termed cardiovascular patterns (in red), group 2 is termed is neuropsychiatric pattern (in yellow), group 3 is termed respiratory-digestive pattern (in green), group 4 is termed metabolic-pain pattern (in blue).

Figure 4: Estimated network of chronic diseases with multimorbidity patterns and gatekeepers diseases (in bold black ring), females aged 80+ at 2011 census, in 2011, 2016 and 2019

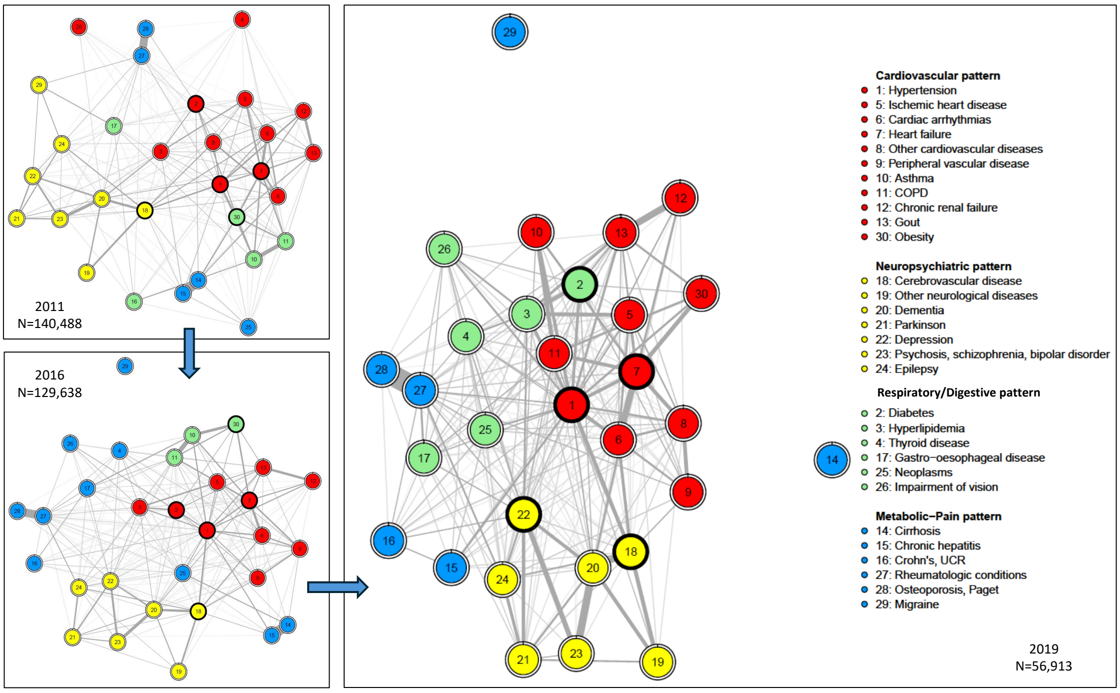

Notes: For the ease of interpretation and the comparability with results from previous research on similar population study by Fortuna et al. (2021), group 1 is termed cardiovascular patterns (in red), group 2 is termed is neuropsychiatric pattern (in yellow), group 3 is termed respiratory-digestive pattern (in green), group 4 is termed metabolic-pain pattern (in blue).

Figure 5: Estimated network of chronic diseases with multimorbidity patterns and gatekeepers diseases (in bold black ring), males aged 50-59 at 2011 census, in 2011, 2016 and 2019

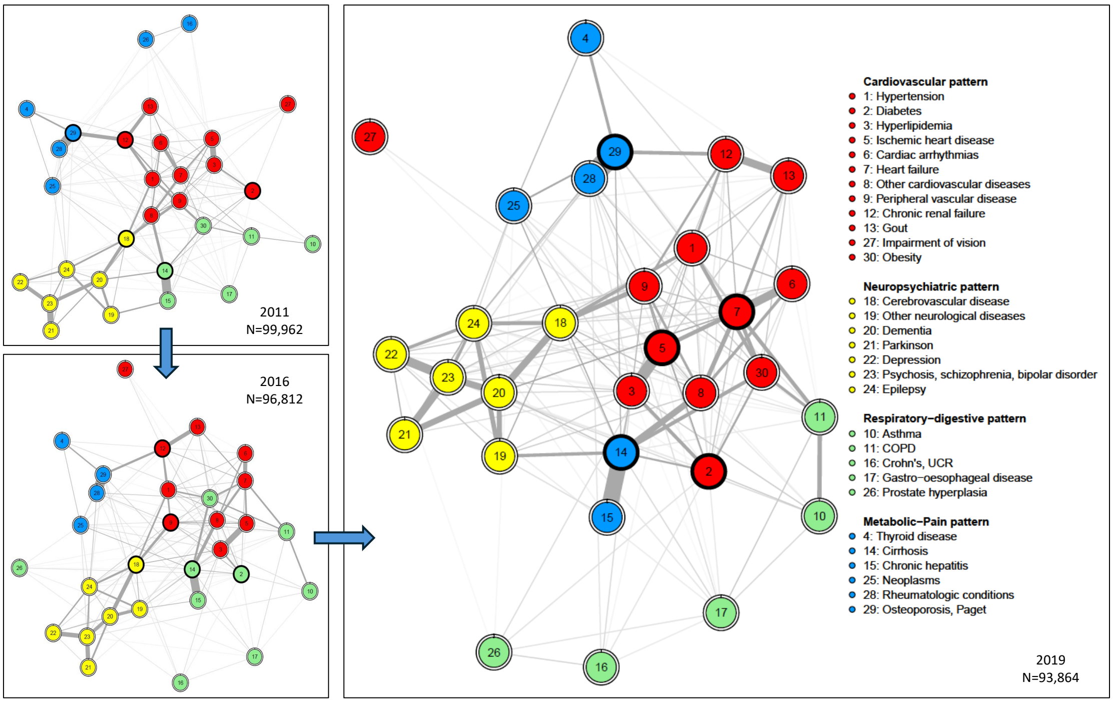

Notes: For the ease of interpretation and the comparability with results from previous research on similar population study by Fortuna et al. (2021), group 1 is termed cardiovascular patterns (in red), group 2 is termed is neuropsychiatric pattern (in yellow), group 3 is termed respiratory-digestive pattern (in green), group 4 is termed metabolic-pain pattern (in blue).

Figure 6: Estimated network of chronic diseases with multimorbidity patterns and gatekeepers diseases (in bold black ring), males aged 60-69 at 2011 census, in 2011, 2016 and 2019

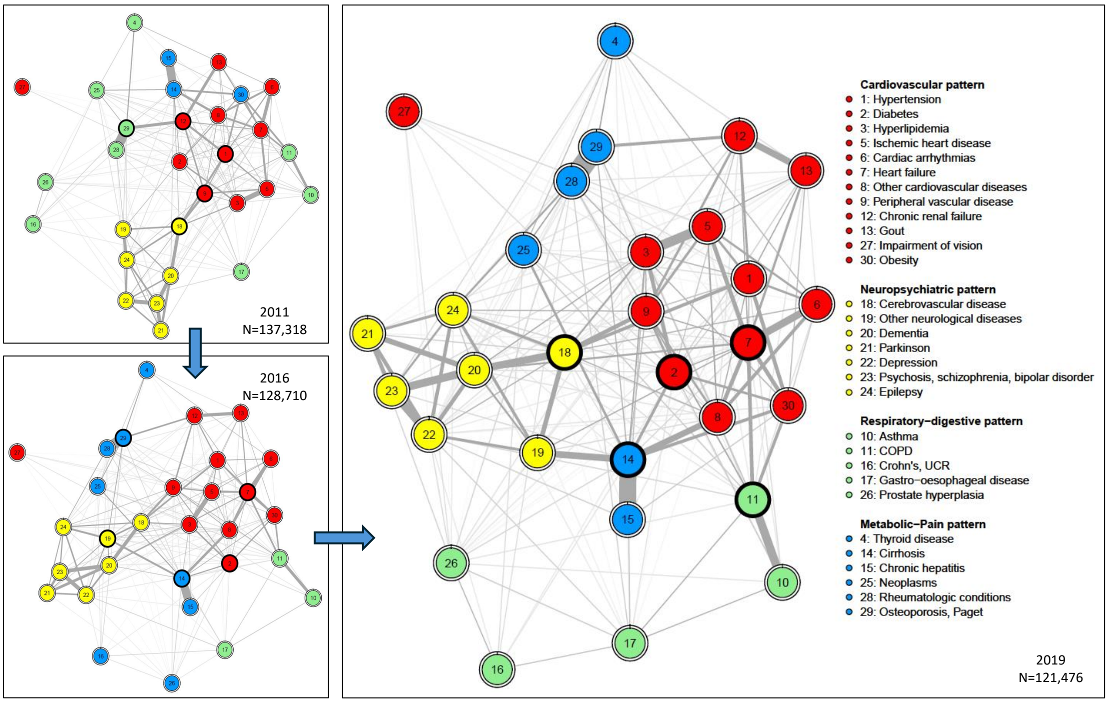

Notes: For the ease of interpretation and the comparability with results from previous research on similar population study by Fortuna et al. (2021), group 1 is termed cardiovascular patterns (in red), group 2 is termed is neuropsychiatric pattern (in yellow), group 3 is termed respiratory-digestive pattern (in green), group 4 is termed metabolic-pain pattern (in blue).

Figure 7: Estimated network of chronic diseases with multimorbidity patterns and gatekeepers diseases (in bold black ring), males aged 70-79 at 2011 census, in 2011, 2016 and 2019

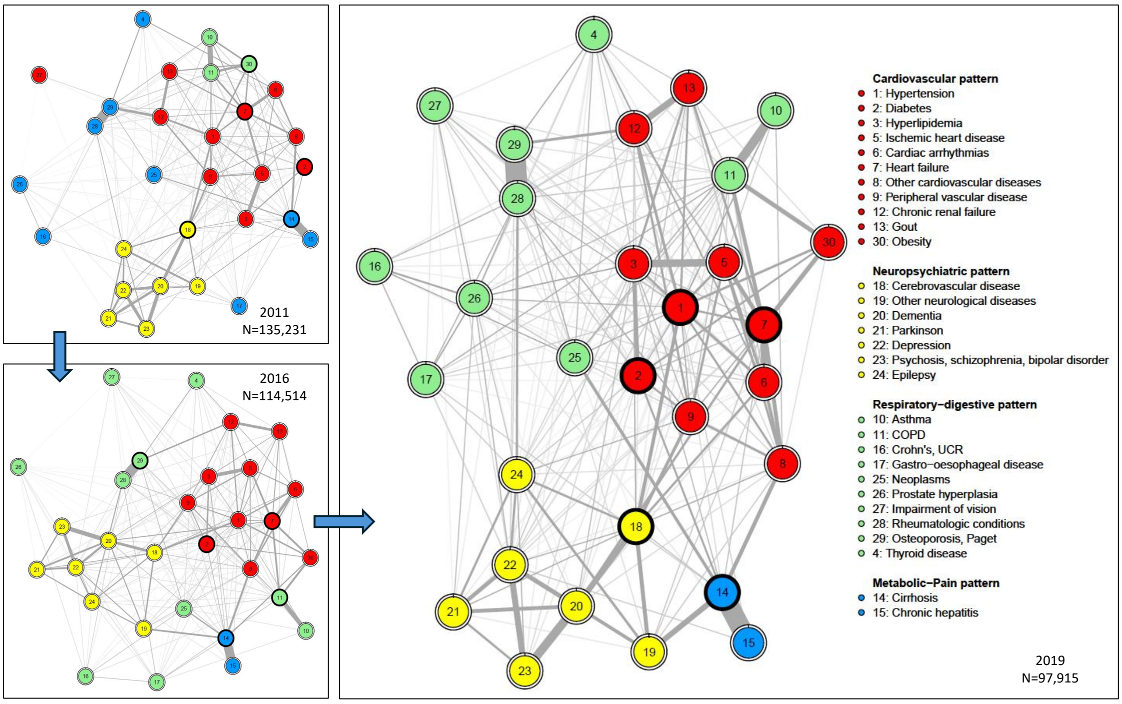

Notes: For the ease of interpretation and the comparability with results from previous research on similar population study by Fortuna et al. (2021), group 1 is termed cardiovascular patterns (in red), group 2 is termed is neuropsychiatric pattern (in yellow), group 3 is termed respiratory-digestive pattern (in green), group 4 is termed metabolic-pain pattern (in blue).

Figure 8: Estimated network of chronic diseases with multimorbidity patterns and gatekeepers diseases (in bold black ring), males aged 80+ at 2011 census, in 2011, 2016 and 2019

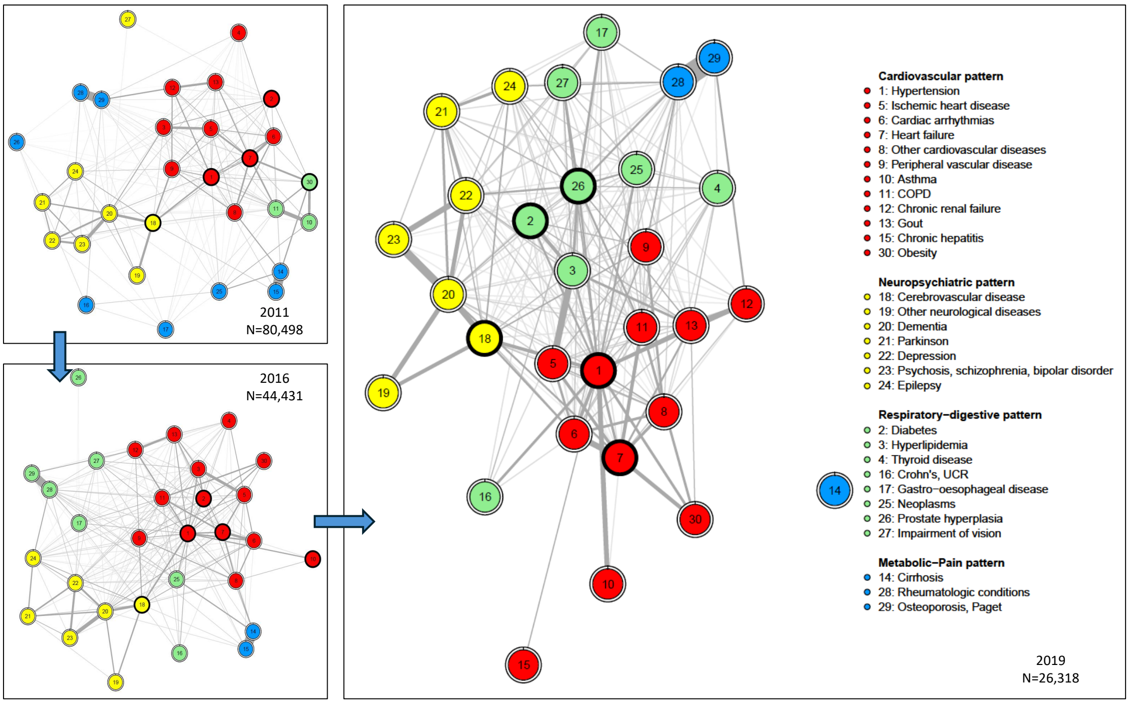

Notes: For the ease of interpretation and the comparability with results from previous research on similar population study by Fortuna et al. (2021), group 1 is termed cardiovascular patterns (in red), group 2 is termed is neuropsychiatric pattern (in yellow), group 3 is termed respiratory-digestive pattern (in green), group 4 is termed metabolic-pain pattern (in blue).
