# Supplementary material for: Population-level analysis of chronic disease multimorbidity at older ages across time using mixed graphical models
Source: Arch Public Health. 2026 Mar 17;84:87. doi: 10.1186/s13690-026-01886-3 (PMC13107661; doi:10.1186/s13690-026-01886-3)
Supplement: Supplementary file 2 — Supplementary Material 2. [file 13690_2026_1886_MOESM2_ESM.docx]

# Appendix

## Chronic diseases prevalence

Figure A1: Chronic diseases prevalence (pooled by sex) across observation period 2011-2019

| **Females** | % |  | **Males** | % |
| --- | --- | --- | --- | --- |
| Hypertension | 45.30 |  | Hypertension | 50.90 |
| Diabetes mellitus | 20.00 |  | Diabetes mellitus | 27.30 |
| Hyperlipidemia | 45.00 |  | Hyperlipidemia | 50.90 |
| Thyroid diseases | 24.40 |  | Thyroid diseases | 8.42 |
| Ischemic heart disease | 14.50 |  | Ischemic heart disease | 25.00 |
| Cardiac arrhythmias | 18.90 |  | Cardiac arrhythmias | 23.30 |
| Heart failure | 15.40 |  | Heart failure | 15.70 |
| Other cardiovascular diseases | 17.10 |  | Other cardiovascular diseases | 16.90 |
| Peripheral vascular disease | 2.71 |  | Peripheral vascular disease | 4.72 |
| Asthma | 2.04 |  | Asthma | 1.44 |
| COPD | 20.20 |  | COPD | 23.70 |
| Chronic renal failure | 1.93 |  | Chronic renal failure | 3.90 |
| Gout | 12.80 |  | Gout | 21.10 |
| Cirrhosis | 2.13 |  | Cirrhosis | 3.00 |
| Chronic hepatitis | 3.14 |  | Chronic hepatitis | 3.71 |
| Crohn's, UCR | 3.13 |  | Crohn's, UCR | 4.12 |
| Gastro-esophageal disease | 14.10 |  | Gastro-esophageal disease | 12.10 |
| Cerebrovascular disease | 16.50 |  | Cerebrovascular disease | 18.40 |
| Other neurological diseases | 1.00 |  | Other neurological diseases | 1.13 |
| Dementia | 15.20 |  | Dementia | 10.70 |
| Parkinson's disease | 4.35 |  | Parkinson's disease | 4.85 |
| Depression | 32.00 |  | Depression | 18.70 |
| Psychosis, schizophrenia, bipolar disorder | 7.15 |  | Psychosis, schizophrenia, bipolar disorder | 5.34 |

*Notes*: The chronic conditions that are marked in bold italic have pooled prevalence across the observation period less than 1% and are excluded from the study, resulting in a set of 30 chronic conditions for each female and male population that are considered systematically for all analyses.

| Epilepsy | 9.97 |
| --- | --- |
| Neoplasms | 34.10 |
| Prostate hyperplasia | 42.70 |
| ***HIV*** | 0.20 |
| Impairment of vision | 12.90 |
| ***Impairment of hearing*** | 0.73 |
| Rheumatologic conditions | 34.30 |
| Osteoporosis, Paget's | 4.89 |
| ***Migraine*** | 0.50 |
| Obesity | 1.03 |

| Epilepsy | 11.90 |
| --- | --- |
| Neoplasms | 27.40 |
| ***HIV*** | ***0.05*** |
| Impairment of vision | 13.10 |
| ***Impairment of hearing*** | ***0.65*** |
| Rheumatologic conditions | 56.50 |
| Osteoporosis, Paget's | 25.90 |
| Migraine | 1.35 |
| Obesity | 1.49 |

Figure A2: Chronic diseases prevalence by age groups for females across observation period 2011-2019

| **Females 50-59** | % |
| --- | --- |
| Hypertension | 25.30 |
| Diabetes mellitus | 14.60 |
| Hyperlipidemia | 38.80 |
| Thyroid diseases | 31.30 |
| Ischemic heart disease | 4.07 |
| Cardiac arrhythmias | 4.39 |
| Heart failure | 1.78 |
| Other cardiovascular diseases | 10.00 |
| Peripheral vascular disease | 0.76 |
| Asthma | 3.66 |
| COPD | 15.60 |
| Chronic renal failure | 0.97 |
| Gout | 4.70 |
| Cirrhosis | 1.82 |
| Chronic hepatitis | 3.10 |
| Crohn's, UCR | 3.38 |
| Gastro-oesophageal disease | 14.50 |
| Cerebrovascular disease | 4.08 |
| Other neurological diseases | 1.21 |
| Dementia | 0.76 |
| Parkinson's disease | 1.61 |
| Depression | 28.50 |
| Psychosis, schizophrenia, bipolar disorder | 5.55 |
| Epilepsy | 10.50 |
| Neoplasms | 25.90 |
| HIV | 0.14 |
| Impairment of vision | 7.20 |
| Impairment of hearing | 0.74 |
| Rheumatologic conditions | 55.60 |
| Osteoporosis, Paget's | 20.90 |
| Migraine | 3.80 |
| Obesity | 2.39 |

| **Females 60-69** | % |
| --- | --- |
| Hypertension | 37.40 |
| Diabetes mellitus | 20.00 |
| Hyperlipidemia | 52.80 |
| Thyroid diseases | 27.90 |
| Ischemic heart disease | 7.68 |
| Cardiac arrhythmias | 9.60 |
| Heart failure | 4.79 |
| Other cardiovascular diseases | 12.70 |
| Peripheral vascular disease | 1.38 |
| Asthma | 2.41 |
| COPD | 18.60 |
| Chronic renal failure | 1.31 |
| Gout | 9.44 |
| Cirrhosis | 1.90 |
| Chronic hepatitis | 3.05 |
| Crohn's, UCR | 3.49 |
| Gastro-oesophageal disease | 16.00 |
| Cerebrovascular disease | 7.51 |
| Other neurological diseases | 0.89 |
| Dementia | 3.29 |
| Parkinson's disease | 2.90 |
| Depression | 27.00 |
| Psychosis, schizophrenia, bipolar disorder | 4.79 |
| Epilepsy | 11.40 |
| Neoplasms | 28.90 |
| HIV | 0.05 |
| Impairment of vision | 12.30 |
| Impairment of hearing | 0.69 |
| Rheumatologic conditions | 59.80 |
| Osteoporosis, Paget's | 26.60 |
| Migraine | 1.37 |
| Obesity | 1.67 |

| **Females 70-79** | % |  | **Females 80+** | % |
| --- | --- | --- | --- | --- |
| Hypertension | 51.90 |  | Hypertension | 61.60 |
| Diabetes mellitus | 22.90 |  | Diabetes mellitus | 20.60 |
| Hyperlipidemia | 53.50 |  | Hyperlipidemia | 32.30 |
| Thyroid diseases | 23.00 |  | Thyroid diseases | 17.00 |
| Ischemic heart disease | 15.50 |  | Ischemic heart disease | 28.80 |
| Cardiac arrhythmias | 21.20 |  | Cardiac arrhythmias | 37.50 |
| Heart failure | 14.90 |  | Heart failure | 37.70 |
| Other cardiovascular diseases | 18.70 |  | Other cardiovascular diseases | 25.50 |
| Peripheral vascular disease | 2.98 |  | Peripheral vascular disease | 5.34 |
| Asthma | 1.52 |  | Asthma | 0.65 |
| COPD | 20.70 |  | COPD | 25.10 |
| Chronic renal failure | 2.13 |  | Chronic renal failure | 3.14 |
| Gout | 16.30 |  | Gout | 18.60 |
| Cirrhosis | 2.50 |  | Cirrhosis | 2.18 |
| Chronic hepatitis | 3.60 |  | Chronic hepatitis | 2.74 |
| Crohn's, UCR | 3.31 |  | Crohn's, UCR | 2.35 |
| Gastro-oesophageal disease | 14.90 |  | Gastro-oesophageal disease | 11.00 |
| Cerebrovascular disease | 18.00 |  | Cerebrovascular disease | 34.10 |
| Other neurological diseases | 1.02 |  | Other neurological diseases | 0.60 |
| Dementia | 15.10 |  | Dementia | 36.20 |
| Parkinson's disease | 5.80 |  | Parkinson's disease | 6.38 |
| Depression | 33.90 |  | Depression | 38.00 |
| Psychosis, schizophrenia, bipolar  disorder | 7.22 |  | Psychosis, schizophrenia, bipolar  disorder | 10.80 |
| Epilepsy | 13.90 |  | Epilepsy | 11.50 |
| Neoplasms | 29.40 |  | Neoplasms | 24.80 |
| HIV | 0.03 |  | HIV | 0.01 |
| Impairment of vision | 16.10 |  | Impairment of vision | 15.40 |
| Impairment of hearing | 0.60 |  | Impairment of hearing | 0.61 |
| Rheumatologic conditions | 61.10 |  | Rheumatologic conditions | 48.70 |
| Osteoporosis, Paget's | 29.70 |  | Osteoporosis, Paget's | 24.60 |
| Migraine | 0.59 |  | Migraine | 0.23 |
| Obesity | 1.33 |  | Obesity | 0.76 |

Figure A3: Chronic diseases prevalence by age groups for males across observation period 2011-2019

| **Males 50-59** | % | **Males 60-69** | % |  | **Males 70-79** | % |  | **Males 80+** | % |
| --- | --- | --- | --- | --- | --- | --- | --- | --- | --- |
| Hypertension | 37.60 | Hypertension | 47.80 |  | Hypertension | 57.00 |  | Hypertension | 62.00 |
| Diabetes mellitus | 25.50 | Diabetes mellitus | 26.00 |  | Diabetes mellitus | 28.60 |  | Diabetes mellitus | 23.70 |
| Hyperlipidemia | 50.10 | Hyperlipidemia | 56.60 |  | Hyperlipidemia | 54.10 |  | Hyperlipidemia | 36.10 |
| Thyroid diseases | 8.38 | Thyroid diseases | 8.46 |  | Thyroid diseases | 8.57 |  | Thyroid diseases | 8.07 |
| Ischemic heart disease | 15.80 | Ischemic heart disease | 21.20 |  | Ischemic heart disease | 28.40 |  | Ischemic heart disease | 37.30 |
| Cardiac arrhythmias | 8.76 | Cardiac arrhythmias | 15.70 |  | Cardiac arrhythmias | 26.10 |  | Cardiac arrhythmias | 44.60 |
| Heart failure | 4.10 | Heart failure | 7.56 |  | Heart failure | 18.50 |  | Heart failure | 38.70 |
| Other cardiovascular diseases | 11.10 | Other cardiovascular diseases | 13.80 |  | Other cardiovascular diseases | 16.60 |  | Other cardiovascular diseases | 24.80 |
| Peripheral vascular disease | 1.65 | Peripheral vascular disease | 3.56 |  | Peripheral vascular disease | 6.07 |  | Peripheral vascular disease | 7.85 |
| Asthma | 2.58 | Asthma | 1.56 |  | Asthma | 0.65 |  | Asthma | 0.61 |
| COPD | 14.20 | COPD | 16.50 |  | COPD | 27.40 |  | COPD | 36.20 |
| Chronic renal failure | 2.21 | Chronic renal failure | 3.28 |  | Chronic renal failure | 4.70 |  | Chronic renal failure | 5.70 |
| Gout | 13.60 | Gout | 16.60 |  | Gout | 25.30 |  | Gout | 25.50 |
| Cirrhosis | 3.22 | Cirrhosis | 2.60 |  | Cirrhosis | 3.16 |  | Cirrhosis | 2.56 |
| Chronic hepatitis | 4.84 | Chronic hepatitis | 3.56 |  | Chronic hepatitis | 3.56 |  | Chronic hepatitis | 2.70 |
| Crohn's, UCR | 4.85 | Crohn's, UCR | 4.66 |  | Crohn's, UCR | 3.66 |  | Crohn's, UCR | 2.53 |
| Gastro-esophageal disease | 11.60 | Gastro-esophageal disease | 12.20 |  | Gastro-esophageal disease | 12.80 |  | Gastro-esophageal disease | 11.30 |
| Cerebrovascular disease | 7.17 | Cerebrovascular disease | 11.60 |  | Cerebrovascular disease | 23.00 |  | Cerebrovascular disease | 35.70 |
| Other neurological diseases | 1.14 | Other neurological diseases | 1.05 |  | Other neurological diseases | 1.22 |  | Other neurological diseases | 1.12 |
| Dementia | 1.03 | Dementia | 3.40 |  | Dementia | 13.00 |  | Dementia | 31.30 |
| Parkinson's disease | 1.75 | Parkinson's disease | 3.28 |  | Parkinson's disease | 6.86 |  | Parkinson's disease | 7.68 |
| Depression | 15.50 | Depression | 14.80 |  | Depression | 20.50 |  | Depression | 26.70 |
| Psychosis, schizophrenia, bipolar disorder | 4.76 | Psychosis, schizophrenia, bipolar disorder | 3.66 |  | Psychosis, schizophrenia, bipolar  disorder | 5.58 |  | Psychosis, schizophrenia, bipolar  disorder | 8.48 |
| Epilepsy | 8.62 | Epilepsy | 6.34 |  |  |  |  |  |  |
| Neoplasms | 22.40 | Neoplasms | 33.10 |  | Epilepsy | 11.40 |  | Epilepsy | 6.65 |
| Prostate hyperplasia | 25.20 | Prostate hyperplasia | 42.60 |  | Neoplasms | 40.80 |  | Neoplasms | 36.00 |
| HIV | 0.54 | HIV | 0.17 |  | Prostate hyperplasia | 51.40 |  | Prostate hyperplasia | 46.50 |
| Impairment of vision | 7.48 | Impairment of vision | 11.60 |  | HIV | 0.06 |  | HIV | 0.03 |
| Impairment of hearing | 0.77 | Impairment of hearing | 0.74 |  | Impairment of vision | 15.80 |  | Impairment of vision | 16.80 |
| Rheumatologic conditions | 36.60 | Rheumatologic conditions | 35.00 |  | Impairment of hearing | 0.68 |  | Impairment of hearing | 0.73 |
| Osteoporosis, Paget's | 2.76 | Osteoporosis, Paget's | 3.88 |  | Rheumatologic conditions | 34.80 |  | Rheumatologic conditions | 26.50 |
| Migraine | 1.16 | Migraine | 0.52 |  | Osteoporosis, Paget's | 5.66 |  | Osteoporosis, Paget's | 7.45 |
| Obesity | 1.62 | Obesity | 1.17 |  | Migraine | 0.21 |  | Migraine | 0.12 |
|  | | | | | Obesity | 0.82 |  | Obesity | 0.38 |

## Impact of the multimorbidity patterns on mortality at older ages

Figure A4: Estimated hazard ratio using multimorbidity patterns as time- dependent predictor for mortality, females aged 50-59 at census 2011, fol- lowed up to 2019

Figure A5: Estimated hazard ratio using multimorbidity patterns as time- dependent predictor for mortality, females aged 60-69 at census 2011, fol- lowed up to 2019

Hazard ratio Hazard ratio

**pattern**

1

*(N=166250)*

reference

2 1.46

*(N=43402) (1.36 − 1.57)*

*<0.001 ****

3

*(N=25672)*

1.17

*(1.05 − 1.30)*

*0.004 ***

4

*(N=91827)*

1.07

*(1.00 − 1.14)*

*0.043 **

**totalpat**

*(N=327151)*

1.23

*(1.22 − 1.25)*

*<0.001 ****

**educ2**

1

*(N=49464)*

reference

2

*(N=157454)*

0.91

*(0.84 − 0.97)*

*0.007 ***

3

*(N=81033)*

0.89

*(0.82 − 0.96)*

*0.004 ***

4

*(N=39200)*

0.81

*(0.73 − 0.89)*

*<0.001 ****

**degurban**

1

*(N=111263)*

reference

2

*(N=146132)*

0.86

*(0.81 − 0.91)*

*<0.001 ****

3

*(N=69756)*

0.93

*(0.86 − 1.00)*

*0.036 **

*# Events: 5609; Global p−value (Log−Rank): 0 AIC: 119917.6; Concordance Index: 0.67*

**pattern**

1

*(N=242204)*

reference

2 1.27

*(N=45084) (1.21 − 1.32)*

*<0.001 ****

3

*(N=24298)*

1.27

*(1.18 − 1.37)*

*<0.001 ****

4

*(N=110364)*

1.13

*(1.09 − 1.18)*

*<0.001 ****

**totalpat**

*(N=421950)*

1.22

*(1.22 − 1.23)*

*<0.001 ****

**educ2**

1

*(N=202746)*

reference

2

*(N=136909)*

0.94

*(0.90 − 0.97)*

*<0.001 ****

3

*(N=52499)*

1.00

*(0.95 − 1.06)*

*0.935*

4

*(N=29796)*

0.85

*(0.79 − 0.91)*

*<0.001 ****

**degurban**

1

*(N=155610)*

reference

2

*(N=183092)*

0.94

*(0.90 − 0.97)*

*<0.001 ****

3

*(N=83248)*

1.05

*(1.00 − 1.10)*

*0.036 **

*# Events: 14182; Global p−value (Log−Rank): 0 AIC: 330901.61; Concordance Index: 0.67*

0.7 0.8 0.9 1 1.1 1.2 1.3 1.4 1.5 1.6 0.8 0.9 1 1.1 1.2 1.3 1.4

*Notes*: The controlled variables are the multimorbidity patterns (*pattern*) (1 = cardiovascular pattern, 2 = neuropsychiatric pattern, 3 = respiratory-digestive pattern, 4 = metabolic-pain pattern), the total number of diagnosed chronic diseases (*totalpat* ), the level of education (*educ2* ) (1 = no education/primary education, 2 = lower secondary education, 3 = upper secondary education, 4 = university), the rural/urban residency (*degurban*) (1 = cities, 2 = small cities or suburbs, 3 = rural areas)

25

*Notes*: The controlled variables are the multimorbidity patterns (*pattern*) (1 = cardiovascular pattern, 2 = neuropsychiatric pattern, 3 = respiratory-digestive pattern, 4 = metabolic-pain pattern), the total number of diagnosed chronic diseases (*totalpat* ), the level of education (*educ2* ) (1 = no education/primary education, 2 = lower secondary education, 3 = upper secondary education, 4 = university), the rural/urban residency (*degurban*) (1 = cities, 2 = small cities or suburbs, 3 = rural areas)

Figure A6: Estimated hazard ratio using multimorbidity patterns as time- dependent predictor for mortality, females aged 70-79 at census 2011, fol- lowed up to 2019

Hazard ratio

Figure A7: Estimated hazard ratio using multimorbidity patterns as time- dependent predictor for mortality, females aged 80+ at census 2011, followed up to 2019

Hazard ratio

**pattern**

1

*(N=162611)*

reference

*****

3 0.88

*(N=16277) (0.85 − 0.91)*

*<0.001 ****

*****

**totalpat**

*(N=269760)*

1.04

*(1.04 − 1.04)*

*<0.001 ****

2 0.88

*(N=29606) (0.86 − 0.90)*

*<0.001 ****

*****

4

*(N=6041)*

0.87

*(0.83 − 0.91)*

*<0.001 ****

2

*(N=106183)*

1.02

*(1.00 − 1.03)*

*0.035 **

*****

*# Events: 100897; Global p−value (Log−Rank): 0 AIC: 2238960.01; Concordance Index: 0.58*

*<0.001*

*(N=57297) (1.08 − 1.11)*

1.09

3

reference

1

*(N=106280)*

**degurban**

*<0.001*

*(N=14300) (0.85 − 0.90)*

0.87

3

reference

1

*(N=219813)*

**educ2**

*<0.001*

*(N=42806) (0.66 − 0.68)*

0.67

4

*<0.001*

*(N=48066) (1.42 − 1.46)*

1.44

2

**pattern**

1

*(N=254045)*

reference

2 1.32

*(N=70646) (1.29 − 1.35)*

*<0.001 ****

3

*(N=20988)*

0.71

*(0.67 − 0.76)*

*<0.001 ****

4

*(N=79142)*

1.12

*(1.09 − 1.15)*

*<0.001 ****

**totalpat**

*(N=424821)*

1.17

*(1.17 − 1.17)*

*<0.001 ****

**educ2**

1

*(N=307616)*

reference

2

*(N=74010)*

0.92

*(0.90 − 0.95)*

*<0.001 ****

3

*(N=30869)*

0.92

*(0.88 − 0.96)*

*<0.001 ****

4

*(N=12326)*

1.01

*(0.95 − 1.08)*

*0.687*

**degurban**

1

*(N=165027)*

reference

2

*(N=176613)*

1.05

*(1.02 − 1.07)*

*<0.001 ****

3

*(N=83181)*

1.15

*(1.12 − 1.18)*

*<0.001 ****

*# Events: 41431; Global p−value (Log−Rank): 0 AIC: 976944.1; Concordance Index: 0.65*

0.7 0.8 0.9 1 1.1 1.2 1.3 1.4 1.5

0.7 0.8 0.9 1 1.1 1.2 1.3 1.4

26

*Notes*: The controlled variables are the multimorbidity patterns (*pattern*) (1 = cardiovascular pattern, 2 = neuropsychiatric pattern, 3 = respiratory-digestive pattern, 4 = metabolic-pain pattern), the total number of diagnosed chronic diseases (*totalpat* ), the level of education (*educ2* ) (1 = no education/primary education, 2 = lower secondary education, 3 = upper secondary education, 4 = university), the rural/urban

*Notes*: The controlled variables are the multimorbidity patterns (*pattern*) (1 = cardiovascular pattern, 2 = neuropsychiatric pattern, 3 = respiratory-digestive pattern, 4 = metabolic-pain pattern), the total number of diagnosed chronic diseases (*totalpat* ), the level of education (*educ2* ) (1 = no education/primary education, 2 = lower secondary education, 3 = upper secondary education, 4 = university), the rural/urban residency (*degurban*) (1 = cities, 2 = small cities or suburbs, 3 = rural areas)

Figure A8: Estimated hazard ratio using multimorbidity patterns as time- dependent predictor for mortality, males aged 50-59 at census 2011, followed up to 2019

Hazard ratio

Figure A9: Estimated hazard ratio using multimorbidity patterns as time- dependent predictor for mortality, males aged 60-69 at census 2011, followed up to 2019

Hazard ratio

**pattern**

1

*(N=201128)*

reference

2 1.83

*(N=20904) (1.71 − 1.95)*

*<0.001*

3

*(N=27106)*

1.57

*(1.46 − 1.68)*

*<0.001*

4

*(N=39952)*

1.59

*(1.49 − 1.69)*

*<0.001*

**totalpat**

*(N=289090)*

1.23

*(1.22 − 1.24)*

*<0.001*

**educ2**

1

*(N=36108)*

reference

2

*(N=137582)*

0.78

*(0.74 − 0.83)*

*<0.001*

3

*(N=81451)*

0.65

*(0.61 − 0.69)*

*<0.001*

4

*(N=33949)*

0.58

*(0.53 − 0.64)*

*<0.001*

**degurban**

1

*(N=94884)*

reference

2

*(N=129556)*

0.91

*(0.87 − 0.96)*

*<0.001*

3

*(N=64650)*

1.08

*(1.02 − 1.14)*

*0.009 ***

*# Events: 8365; Global p−value (Log−Rank): 0 AIC: 188764.71; Concordance Index: 0.68*

*****

**pattern**

1

*(N=265859)*

reference

2 1.62

*(N=24890) (1.55 − 1.69)*

*<0.001*

3

*(N=52587)*

2.08

*(2.00 − 2.17)*

*<0.001*

4

*(N=40309)*

0.96

*(0.91 − 1.00)*

*0.06*

**totalpat**

*(N=383645)*

1.20

*(1.20 − 1.21)*

*<0.001*

**educ2**

1

*(N=135913)*

reference

2

*(N=138669)*

0.85

*(0.83 − 0.88)*

*<0.001*

3

*(N=71824)*

0.81

*(0.78 − 0.84)*

*<0.001*

4

*(N=37239)*

0.68

*(0.64 − 0.72)*

*<0.001*

**degurban**

1

*(N=132067)*

reference

2

*(N=168500)*

0.96

*(0.93 − 0.99)*

*0.005 ***

3

*(N=83078)*

1.05

*(1.01 − 1.09)*

*0.006 ***

*# Events: 21934; Global p−value (Log−Rank): 0 AIC: 510242.64; Concordance Index: 0.69*

*****

*****

*****

*****

*****

*****

*****

*****

*****

*****

*****

*****

*****

0.8 1 1.2 1.4 1.6 1.8 2 2.2 2.4

0.6 0.8 1 1.2 1.4 1.6 1.8 2

27

*Notes*: The controlled variables are the multimorbidity patterns (*pattern*) (1 = cardiovascular pattern, 2 = neuropsychiatric pattern, 3 = respiratory-digestive pattern, 4 = metabolic-pain pattern), the total number of diagnosed chronic diseases (*totalpat* ), the level of education (*educ2* ) (1 = no education/primary education, 2 = lower secondary education, 3 = upper secondary education, 4 = university), the rural/urban

*Notes*: The controlled variables are the multimorbidity patterns (*pattern*) (1 = cardiovascular pattern, 2 = neuropsychiatric pattern, 3 = respiratory-digestive pattern, 4 = metabolic-pain pattern), the total number of diagnosed chronic diseases (*totalpat* ), the level of education (*educ2* ) (1 = no education/primary education, 2 = lower secondary education, 3 = upper secondary education, 4 = university), the rural/urban

residency (*degurban*) (1 = cities, 2 = small cities or suburbs, 3 = rural areas)

Figure A10: Estimated hazard ratio using multimorbidity patterns as time- dependent predictor for mortality, males aged 70-79 at census 2011, followed up to 2019

Figure A11: Estimated hazard ratio using multimorbidity patterns as time- dependent predictor for mortality, males aged 80+ at census 2011, followed up to 2019

Hazard ratio Hazard ratio

*****

**pattern**

1

*(N=225579)*

reference

*<0.001*

3

*(N=55023)*

0.79

*(0.77 − 0.82)*

*<0.001*

*<0.001*

**totalpat**

*(N=339054)*

1.15

*(1.15 − 1.15)*

*<0.001*

2

*(N=79865)*

0.86

*(0.84 − 0.87)*

*<0.001*

*<0.001*

4

*(N=19766)*

0.75

*(0.72 − 0.79)*

*<0.001*

2 1.01

*(N=144574) (0.99 − 1.03)*

*<0.001*

*# Events: 50481; Global p−value (Log−Rank): 0 AIC: 1178927.04; Concordance Index: 0.67*

3 1.12

*(N=70956) (1.09 − 1.15)*

*0.266*

reference

1

*(N=123524)*

**degurban**

3 0.82

*(N=36565) (0.80 − 0.85)*

reference

1

*(N=202858)*

**educ2**

4 2.57

*(N=31998) (2.49 − 2.65)*

2 1.61

*(N=26454) (1.57 − 1.65)*

**pattern**

1

*(N=90674)*

reference

2 1.48

*(N=18400) (1.45 − 1.51)*

*<0.001 ****

3

*(N=18778)*

0.83

*(0.81 − 0.86)*

*<0.001 ****

4

*(N=14887)*

0.80

*(0.78 − 0.82)*

*<0.001 ****

**totalpat**

*(N=142739)*

1.04

*(1.04 − 1.04)*

*<0.001 ****

**educ2**

1

*(N=100854)*

reference

2

*(N=23664)*

0.91

*(0.89 − 0.93)*

*<0.001 ****

3

*(N=10989)*

0.88

*(0.85 − 0.90)*

*<0.001 ****

4

*(N=7232)*

0.91

*(0.88 − 0.95)*

*<0.001 ****

**degurban**

1

*(N=53738)*

reference

2

*(N=58016)*

1.00

*(0.98 − 1.02)*

*0.927*

3

*(N=30985)*

1.08

*(1.06 − 1.10)*

*<0.001 ****

*# Events: 63325; Global p−value (Log−Rank): 0 AIC: 1332615.46; Concordance Index: 0.56*

*****

*****

*****

*****

*****

*****

*****

1 1.5 2 2.5 3 0.8 0.9 1 1.1 1.2 1.3 1.4 1.5 1.6

*Notes*: The controlled variables are the multimorbidity patterns (*pattern*) (1 = cardiovascular pattern, 2 = neuropsychiatric pattern, 3 = respiratory-digestive pattern, 4 = metabolic-pain pattern), the total number of diagnosed chronic diseases (*totalpat* ), the level of education (*educ2* ) (1 = no education/primary education, 2 = lower secondary education, 3 = upper secondary education, 4 = university), the rural/urban residency (*degurban*) (1 = cities, 2 = small cities or suburbs, 3 = rural areas)

*Notes*: The controlled variables are the multimorbidity patterns (*pattern*) (1 = cardiovascular pattern, 2 = neuropsychiatric pattern, 3 = respiratory-digestive pattern, 4 = metabolic-pain pattern), the total number of diagnosed chronic diseases (*totalpat* ), the level of education (*educ2* ) (1 = no education/primary education, 2 = lower secondary education, 3 = upper secondary education, 4 = university), the rural/urban residency (*degurban*) (1 = cities, 2 = small cities or suburbs, 3 = rural areas)

28
